# Virus-Mediated Transient Expression Techniques Enable Functional Genomics Studies and Modulations of Betalain Biosynthesis and Plant Height in Quinoa

**DOI:** 10.3389/fpls.2021.643499

**Published:** 2021-03-18

**Authors:** Takuya Ogata, Masami Toyoshima, Chihiro Yamamizo-Oda, Yasufumi Kobayashi, Kenichiro Fujii, Kojiro Tanaka, Tsutomu Tanaka, Hiroharu Mizukoshi, Yasuo Yasui, Yukari Nagatoshi, Nobuyuki Yoshikawa, Yasunari Fujita

**Affiliations:** ^1^Biological Resources and Post-harvest Division, Japan International Research Center for Agricultural Sciences (JIRCAS), Tsukuba, Japan; ^2^Technology Development Group, Actree Corporation, Hakusan, Japan; ^3^Graduate School of Agriculture, Kyoto University, Kyoto, Japan; ^4^Agri-Innovation Center, Iwate University, Morioka, Japan; ^5^Graduate School of Life and Environmental Sciences, University of Tsukuba, Tsukuba, Japan

**Keywords:** apple latent spherical virus, betalain, functional genomics, quinoa, virus-induced gene silencing, virus-mediated gene overexpression

## Abstract

Quinoa (*Chenopodium quinoa*), native to the Andean region of South America, has been recognized as a potentially important crop in terms of global food and nutrition security since it can thrive in harsh environments and has an excellent nutritional profile. Even though challenges of analyzing the complex and heterogeneous allotetraploid genome of quinoa have recently been overcome, with the whole genome-sequencing of quinoa and the creation of genotyped inbred lines, the lack of technology to analyze gene function *in planta* is a major limiting factor in quinoa research. Here, we demonstrate that two virus-mediated transient expression techniques, virus-induced gene silencing (VIGS) and virus-mediated overexpression (VOX), can be used in quinoa. We show that apple latent spherical virus (ALSV) can induce gene silencing of quinoa *phytoene desaturase* (*CqPDS1*) in a broad range of quinoa inbred lines derived from the northern and southern highland and lowland sub-populations. In addition, we show that ALSV can be used as a VOX vector in roots. Our data also indicate that silencing a quinoa 3,4-dihydroxyphenylalanine 4,5-dioxygenase gene (*CqDODA1*) or a cytochrome P450 enzyme gene (*CqCYP76AD1*) inhibits betalain production and that knockdown of a reduced-height gene homolog (*CqRHT1*) causes an overgrowth phenotype in quinoa. Moreover, we show that ALSV can be transmitted to the progeny of quinoa plants. Thus, our findings enable functional genomics in quinoa, ushering in a new era of quinoa research.

## Introduction

Rapid and large-scale changes in environmental and social conditions require rapid and fundamental changes in crop productivity and diversity (Eshed and Lippman, 2019). To withstand the effects of climate change and the population pressures on the global food system, the food supply will need to double by 2050 (Wheeler and Braun, 2013). Achieving global sustainable food production will require that people shift from a livestock-based diet to a plant-based diet (Shepon et al., 2018). This shift will need to be accompanied by an increase in the production of protein-rich crops (Eshed and Lippman, 2019). Of the tens of thousands of edible plants currently in existence, hundreds of species are cultivated around the world, but fewer than a dozen account for the majority of calories consumed (Eshed and Lippman, 2019). Diversifying staple crops is thus another requirement for achieving a food supply that is sustainable, resilient, and suited to local environments (Massawe et al., 2016).

In many parts of the world, underutilized, orphan, and neglected crops are cultivated in marginal lands (Massawe et al., 2016). Among these crops is quinoa (*Chenopodium quinoa* Willd.), an annual protein-rich pseudocereal with excellent nutritional properties and an ability to tolerate the stressful environments typical of the Andean region of South America. The seeds and leaves of quinoa contain a wide variety of minerals, vitamins, fats, dietary fiber, natural antioxidants, and high-quality proteins composed of high levels of essential amino acids (Vega-Gálvez et al., 2010; Nowak et al., 2016; Pathan et al., 2019; Rodríguez et al., 2020). Furthermore, quinoa can tolerate a wide spectrum of abiotic stresses such as drought, high salinity, frost, and low temperatures, making it a highly sustainable crop (Jacobsen et al., 2005; Hariadi et al., 2011; Yasui et al., 2016; Mizuno et al., 2020).

Quinoa was first domesticated at least 7,500 years ago (Dillehay et al., 2007). Although quinoa was a staple food for the indigenous people of the Andes in the Pre-Columbian era (González et al., 2015), the Spanish conquistadors banned quinoa production and consumption in the sixteenth century since it was revered as a sacred “mother grain” used in indigenous rituals (González et al., 2015). Unlike tomato (*Solanum lycopersicum* L.) and potato (*Solanum tuberosum* L.), which are also native to the Andes but spread throughout the world during the Spanish conquest, quinoa remained a neglected crop for almost 500 years, until the latter half of the twentieth century (Gomez-Pando, 2015). In the 1950s, plant virologists began to recognize quinoa as an important indicator plant for plant viruses (Uschdraweit, 1955; Hein, 1957). In the 1970s and 1980s, the National Academy of Sciences of the USA (NAS) and the US National Research Council (NRC) deemed quinoa as an underutilized plant with promising economic value for worldwide cultivation (National Academy of Sciences (NAS), 1975; National Research Council (NRC), 1989). In the 1990s, quinoa was considered as an important candidate crop to be grown as part of the USA’s National Aeronautics and Space Administration (NASA) Controlled Ecological Life Support System (CELSS) for long-term space missions due to its high productivity and desirable nutritional and growth features under controlled environments (Schlick and Bubenheim, 1993, 1996). In the twenty-first century, with the aim of raising public awareness of the nutritional benefits of this durable plant in the context of global food and nutrition security, the United Nations (UN) General Assembly designated 2013 as the “International Year of Quinoa,” in recognition of the ancestral practices of the Andean people, who have managed to preserve quinoa in its natural state as food for present and future generations (Bazile et al., 2016). Quinoa popularity is on the rise and is considered an ideal food source in many countries (Vega-Gálvez et al., 2010). However, while quinoa cultivation is currently being attempted in more than 95 countries around the world, the vast majority of commercial quinoa is produced in Bolivia and Peru (Bazile et al., 2016).

In many countries where quinoa is grown, stem lodging, low tolerance to high temperature stress, adverse effects of prolonged photoperiods, and high susceptibility to pests and diseases have been recognized as major limiting factors preventing the global expansion of quinoa cultivation and effective large-scale cultivation (Schmöckel et al., 2017; Gomez-Pando et al., 2019). Nonetheless, the Altiplano is a major production area supplying the world with high-quality quinoa, despite obstacles such as drought (rainfall below 200∼500 mm per year), high altitude (3,800 meters above sea level), frost, hail, and strong wind, which are only intensifying due climate change (Bonifacio, 2019). Addressing challenges in quinoa production and thereby contributing to local and global food and nutrition security will rely on the ability to genetically improve this crop.

Quinoa is an allotetraploid (2*n* = 4*x* = 36) species with an estimated genome size of approximately 1.5 Gbp (Palomino et al., 2008; Yangquanwei et al., 2013). For many years, molecular analysis of quinoa had been limited by its genome complexity, derived from allotetraploidy and its genetic heterogeneity due to partial outcrossing resulting from the existence of both hermaphrodite and female flowers on the same plant (Maughan et al., 2004; Christensen et al., 2007). Recently, our collaborative group (Yasui et al., 2016) and two other independent groups (Jarvis et al., 2017; Zou et al., 2017) have sequenced the quinoa genome. Subsequently, we have developed more than 130 genotyped quinoa inbred lines suitable for molecular analyses and illustrated the genotype–phenotype relationships among the inbred lines with respect to salt tolerance and key growth traits (Mizuno et al., 2020). Our integrative genome analyses using single-nucleotide polymorphisms (SNPs) indicated that the quinoa lines are divided into three genetic sub-populations, namely, the northern and southern highland and the lowland sub-populations (Mizuno et al., 2020). Nonetheless, since gain- and loss-of-function techniques have not been developed for quinoa, the research community does not have the resources needed to analyze the functions of endogenous genes *in planta* or to conduct functional genomics research in this important crop.

Virus-induced gene silencing (VIGS) is a powerful tool for functional genomics in plants, especially in plant species that are recalcitrant to stable transformation (Baulcombe, 1999; Burch-Smith et al., 2004; Senthil-Kumar and Mysore, 2011; Wang et al., 2016). VIGS is a post-transcriptional gene silencing-based technique used to knockdown the expression of endogenous target genes (Baulcombe, 1999; Burch-Smith et al., 2004; Senthil-Kumar and Mysore, 2011). Although no plant virus vectors have been shown to mediate VIGS in quinoa so far, quinoa has been used as a propagation host for apple latent spherical virus (ALSV), which in turn has been used as a VIGS vector for a wide range of dicot plants (Li et al., 2000; Igarashi et al., 2009; Yamagishi and Yoshikawa, 2009; Ogata et al., 2017). ALSV has icosahedral particles that harbor two single-stranded RNA species, RNA1 and RNA2 (Li et al., 2000). The ALSV-RNA2 VIGS vector can be engineered to carry additional DNA fragments for VIGS analysis (Li et al., 2004; Igarashi et al., 2009).

Here, we demonstrate that two virus-mediated transient expression techniques, VIGS and virus-mediated overexpression (VOX), can be used in quinoa. We show that ALSV induces gene silencing of quinoa *phytoene desaturase* (*CqPDS1*) in a broad range of quinoa inbred lines derived from the northern and southern highland and lowland sub-populations, and that ALSV can be used as a VOX vector in quinoa roots. Furthermore, we demonstrate that silencing a quinoa 3,4-dihydroxyphenylalanine (DOPA) 4,5-dioxygenase gene (*CqDODA1*) or a cytochrome P450 enzyme gene (*CqCYP76AD1*) inhibits betalain production and that knockdown of a reduced-height gene homolog (*CqRHT1*) causes an overgrowth phenotype in quinoa. In addition, we show that ALSV VIGS can be transmitted to the next plant generation in quinoa. Our findings present a strategy for conducting functional genomics in quinoa, opening the door to deciphering the molecular mechanisms of this promising crop with exceptional nutritional value and adaptability to various environments.

## Results

### ALSV Induces Silencing of *CqPDS1* in Quinoa

ALSV can infect quinoa systemically (Li et al., 2000) and thus has been used as a propagation host for the ALSV VIGS vector system (Igarashi et al., 2009). We therefore wondered whether ALSV could be used as a vector to analyze endogenous genes in quinoa. To test whether ALSV could induce endogenous gene silencing in quinoa plants, we first used the *PDS* gene as a visible VIGS indicator. *PDS* encodes a key enzyme in carotenoid biosynthesis and has been widely used as a reporter gene, as silencing of *PDS* induces a characteristic photobleaching phenotype that can readily be detected (Ruiz et al., 1998). Based on the sequence data of putative *PDS* genes in quinoa obtained from public databases, we designed PCR primers in the conserved regions and then detected and named homoeolog *CqPDS1* genes, *CqPDS1A* and *CqPDS1B*, in a standard quinoa inbred line, Kd (Yasui et al., 2016). The cloned coding sequence (CDS) of *CqPDS1A* shared 98.5% identity with that of *CqPDS1B* (Supplementary Figure 1). To construct the ALSV vectors harboring a specific trigger sequence required for VIGS, we then introduced two 300-bp fragments of the *CqPDS1* homoeologs, CqPDSN and CqPDSC, from the highly conserved regions between these two genes (Supplementary Figure 1), into the ALSV-RNA2 vector. We obtained inocula for VIGS analyses, including the packaged viruses of ALSV-CqPDSN, ALSV-CqPDSC, and ALSV-WT, from the uninoculated upper leaves with chlorotic spots of quinoa plants (inbred Iw line) inoculated with ALSV-RNA1 cDNA plasmid combined with ALSV-RNA2 VIGS vectors either harboring or not a specific 300-bp trigger sequence required for VIGS (Supplementary Figure 1).

**FIGURE 1:**
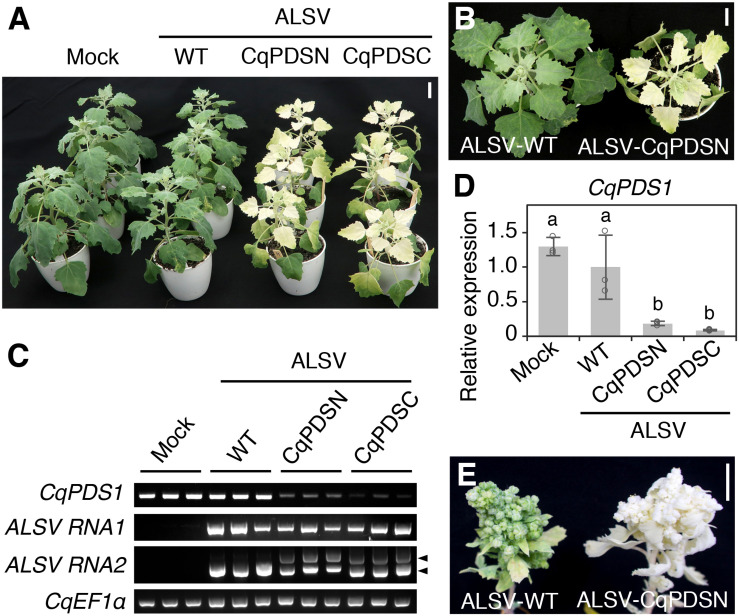
ALSV induces silencing of *CqPDS1* genes in quinoa. **(A)** A representative image of plants (inbred Iw line) at 14 dpi with mock buffer, ALSV-WT, ALSV-CqPDSN, and ALSV-CqPDSC. Three plants were used per inoculum. Scale bar represents 2 cm. **(B)** Top view of representative plants at 14 dpi with ALSV-WT and ALSV-CqPDSN. Scale bar represents 2 cm. **(C)** Semi-quantitative RT-PCR of *CqPDS1*, ALSV RNA1, and RNA2 in the uninoculated upper leaves of plants at 14 dpi with mock, ALSV-WT, ALSV-CqPDSN, and ALSV-CqPDSC. Quinoa *elongation factor 1*α (*CqEF1*α) was used as an internal control. Arrowheads indicate the positions of recombinant ALSV RNA2 with the trigger sequence (top) and WT RNA2 without the trigger sequence (bottom). **(D)** RT-qPCR quantification of *CqPDS1* transcripts in the uninoculated upper leaves of plants inoculated with the indicated inocula. Data were normalized to the *18S ribosomal RNA* (*18S rRNA*) levels and are shown as means ± SD (*n* = 3). Different letters indicate significant differences by a Tukey’s HSD test (*p* < 0.05). **(E)** A representative image of flower buds of quinoa plants at 8 wpi with ALSV-WT and ALSV-CqPDSN. Scale bar represents 1 cm.

Photobleaching phenotypes first appeared in the uninoculated upper leaves of quinoa Iw plants infected with ALSV-CqPDSN or ALSV-CqPDSC at approximately 10 days post inoculation (dpi). By 14 dpi, photobleaching phenotypes were apparent in all upper leaves and stems from the sixth leaves upwards of Iw plants inoculated with ALSV-CqPDSN or ALSV-CqPDSC (Figures 1A,B). Consistently, reverse transcription (RT)-PCR analysis confirmed systemic infection of ALSV and showed that endogenous *CqPDS1* expression was reduced in the uninoculated upper leaves (Figure 1C). Reverse transcription quantitative PCR (RT-qPCR) analysis showed that *CqPDS1* was expressed at less than 20% of the level expressed in plants inoculated with ALSV-WT (Figure 1D). By 8 weeks post inoculation (wpi), photobleaching was observed even in the flower buds of Iw plants inoculated with ALSV-CqPDSN (Figure 1E). Thus, the ALSV vector can induce endogenous gene silencing in quinoa Iw line plants through systemic infection of ALSV.

### The ALSV Vector Is Suitable for Gene Function Analysis of a Variety of Quinoa Inbred Lines

**FIGURE 2:**
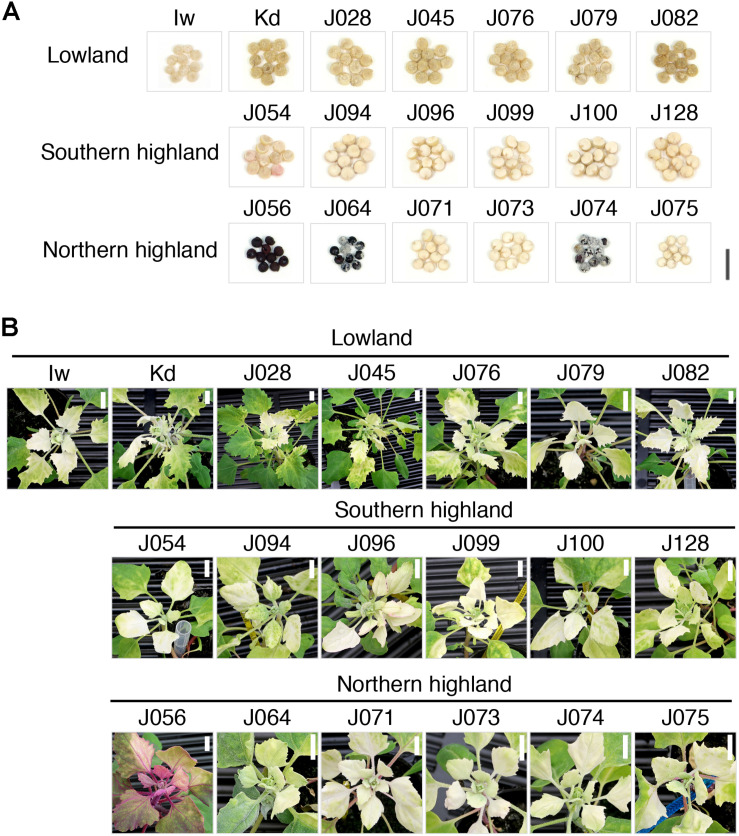
The ALSV vector is suitable for gene function analysis in a variety of quinoa inbred lines. **(A)** Representative images of quinoa seeds of 19 representative inbred lines, including seven lowland, six southern, and six northern highland lines. Each representative inbred line selected in this analysis contains 100% of the genetic background of each sub-population based on the results of our previous GBS analysis of the inbred quinoa lines (Mizuno et al., 2020). Scale bar represents 5 mm. **(B)** Representative images of the 19 representative inbred line plants at 16 dpi with ALSV-CqPDSC. Scale bars represent 1 cm.

Since quinoa line Iw, which we used in the VIGS of *CqPDS1*, belongs to the lowland sub-population, it was unclear whether ALSV could induce endogenous gene silencing in the other genotypes too. We thus tested whether ALSV-CqPDSC could induce silencing of *CqPDS1* in 19 representative lines from the lowland (seven lines), southern highland (six lines), and northern highland (six lines) sub-populations (Table 1 and Figure 2A). Each representative line selected for this analysis contained 100% of the genetic background of each sub-population based on the results of our previous genotyping-by-sequencing (GBS) analysis of the inbred quinoa lines (Mizuno et al., 2020). For all 19 quinoa genotypes, entire photobleaching phenotypes were consistently observed by 16 dpi in the uninoculated upper leaves of quinoa plants inoculated with ALSV-CqPDSC (Figure 2B and Table 1). These findings suggest that ALSV induces gene silencing in a wide range of quinoa lines.

**TABLE 1:** List of quinoa inbred lines used in this study and summary of the occurrence of the photobleaching phenotype in ALSV-CqPDSC-infected plants.

Name of sub-population*	Name of inbred lines*	Accession No. for USDA*	Plant name*	Origin*	No. of plants showing photobleaching phenotype/No. of plants inoculated with ALSV-CqPDSC	Symptoms and phenotypes in ALSV-WT-infected plants at 2–3 wpi	Note
Lowland	Iw	N.A.^†^	Iwate	Japan, Iwate^‡^	17/17	Mild stunting	An inbred line used and appropriate for propagation of ALSV. Characteristic symptoms of chlorotic spots and leaf dwarfing appear during the early infection stage.
	Kd	N.A.^†^	Kyoto-d	Japan, Kyoto^‡^	16/16	Abnormal leaf growth, leaf dwarfing, and stunting	A standard inbred line due to its outstanding stability in phenotypic uniformity; whole-genome sequence was reported in this inbred line (Yasui et al., 2016)
	J028	Ames 13744	409	United States, New Mexico^‡^	14/14	Almost no symptoms and no negative effect on its growth	
	J045	Ames 13761	47TES	United States, New Mexico^‡^	14/14	Almost no symptoms and no negative effect on its growth	
	J076	PI 584524	QQ056	Chile, Chillan	14/14	Mild stunting	
	J079	PI 614882	QQ67	Chile, La Araucania	14/14	Mild mosaic symptom, leaf dwarfing, and stunting	
	J082	PI 614886	QQ74	Chile, Maule	20/20	Almost no symptoms and no negative effect on its growth	Whole-genome sequence was reported in this accession (Jarvis et al., 2017)
Southern highland	J054	PI 478408	R-64	Bolivia, La Paz	11/11	Mild stunting	
	J094	PI 614911	CQ111	Bolivia, Oruro	13/13	Mild stunting	
	J096	PI 614913	CQ113	Bolivia, Oruro	16/16	Mild stunting	
	J099	PI 614916	CQ116	Bolivia, Oruro	14/14	Mild stunting	
	J100	PI 614917	CQ117	Bolivia, Oruro	15/15	Mild stunting	
	J128	PI 643079	Pasankalla	Peru, Puno	14/14	Mild stunting	
	J131	PI 665275	Line 0692	Bolivia, La Paz	N.D.^§^	Enhanced accumulation of betalains and mild stunting	High levels of betalain accumulation
Northern highland	J056	PI 478414	R-70	Bolivia, La Paz	14/14	Enhanced accumulation of betalains and mild stunting	High levels of betalain accumulation
	J064	PI 510537	Koito Jaira (Aymara)	Peru, Puno	14/14	Leaf dwarfing, mosaic, and stunting	
	J071	PI 510546	Jancco Juira (Aymara)	Peru, Puno	15/15	Enhanced accumulation of betalains and mild stunting	
	J073	PI 510548	Yulaj Q’anq’olla (Quechua)	Peru, Puno	13/13	Enhanced accumulation of betalains and mild stunting	
	J074	PI 510549	Yulaj K’oyto (Quechua)	Peru, Puno	17/17	Leaf dwarfing, mosaic, and stunting	
	J075	PI 510551	Quinua (Quechua)	Peru, Puno	14/14	Enhanced accumulation of betalains and stunting	

*See previous report (Mizuno et al., 2020) for details. ^†^N.A., not assigned. ^‡^These accessions lack reliable passport data (Mizuno et al., 2020). ^§^N.D., not determined.

When analyzing the function of plant genes using VIGS, the effects of viral infection on the plant should be kept to a minimum. Therefore, we next inoculated the 19 representative quinoa lines with ALSV-WT and monitored the effect of ALSV infection on the quinoa plants. For most of the quinoa inbred lines examined, very mild mosaic symptoms were apparent in the uninoculated upper leaves of quinoa plants inoculated with ALSV-WT during the early stages of infection (at around 7–10 dpi) but the symptoms were nearly absent by 2–3 wpi (Table 1 and Supplementary Figures 2–4). ALSV RNA was detected in the uninoculated upper leaves of all of the quinoa inbred lines inoculated with ALSV-WT (Supplementary Figure 5), indicating that most of the genotypes have a latent ALSV infection. Three lowland inbred lines, J028, J045, and J082, displayed almost no symptoms, and showed no negative effect on their growth and plant size following inoculation with ALSV-WT (Table 1 and Supplementary Figure 2), suggesting that these three lines are well suited for ALSV VIGS analysis as they are not affected by ALSV infection. Mild stunted growth but no visible symptoms in the uninoculated upper leaves was observed in many quinoa plants of the northern and southern highland sub-populations inoculated with ALSV-WT (Table 1 and Supplementary Figures 3, 4). In addition, ALSV infection caused the red-violet color of the leaves and stems to become darker in the later stages of infection in four northern highland lines, J056, J071, J073, and J075 (Supplementary Figure 6A).

Unlike the other genotypes, the visible symptoms observed in J064, J074, J079, and Kd were sustained for several wpi (Table 1 and Supplementary Figures 2, 4). Particularly, ALSV infection induced stunting, leaf dwarfing, and visible mosaic symptoms in two northern highland inbred lines, J064 and J074 (Table 1 and Supplementary Figure 4), and also induced abnormal leaf growth and leaf dwarfing in the later stages of infection in Kd, a lowland inbred line (Table 1 and Supplementary Figure 2). However, considering that the induction of disease symptoms and VIGS in the leaves of quinoa plants inoculated with ALSV-WT and ALSV-CqPDSC, respectively, was accelerated and enhanced when plants were grown at 25°C compared to 22°C (Supplementary Figure 7), it may be possible to modulate the effects of ALSV infection by adjusting the growth conditions. Thus, these results demonstrate that the ALSV vector system is suitable for gene function analysis of a wide range of quinoa genotypes.

### ALSV Can Be Used as a VOX Vector in Roots

The ALSV vector was used to express exogenous genes in quinoa leaves (Takahashi and Yoshikawa, 2008). To further expand the application of the ALSV vector in quinoa, we next examined whether ALSV could be used as a vector for virus-mediated overexpression (VOX) in roots as well as for VIGS in quinoa leaves. At 2 days post germination (dpg), the roots and hypocotyls of J082 plants, whose growth was not affected by ALSV infection during the vegetative stage, were inoculated with the packaged virus derived from recombinant ALSV harboring a full-length *green fluorescence protein* (*GFP*) CDS, using carborundum. GFP fluorescence and virus accumulation were observed in the roots of quinoa plants at 10 dpi (Supplementary Figure 8), indicating that ALSV can indeed be used as a VOX vector in roots. Hence, these collective results demonstrate that the ALSV could be used as a vector for both VIGS and VOX for gene function analysis in a whole quinoa plant.

### Silencing of *CqDODA1* and *CqCYP76AD1* Inhibits Betalain Production in Quinoa

In plants, three major pigment classes, namely, carotenoids, anthocyanins, and betalains, are generally responsible for the attractive colors observed in fruits, flowers, and vegetative tissues (Tanaka et al., 2008). Most flowering plants produce anthocyanins, whereas functionally equivalent betalains are produced solely by Caryophyllales species such as quinoa (Stafford, 1994; Strack et al., 2003; Brockington et al., 2015). These two classes of pigments are known to be present in a mutually exclusive fashion (Stafford, 1994; Strack et al., 2003; Brockington et al., 2015). Although the biosynthesis of carotenoids and anthocyanins has been well characterized, betalain production and its regulation remain largely unknown (Polturak and Aharoni, 2018). Nevertheless, since betalains have been reported to have strong antioxidant and health-promoting properties, betalains are of scientific interest and may be of economic importance (Polturak and Aharoni, 2018).

Quinoa, beet (*Beta vulgaris* L.), and spinach (*Spinacia oleracea* L.) are Caryophyllales plant species that possess carotenoids and betalains in organs such as the leaves, stems, and seeds (Figure 2A and Supplementary Figures 2–4). To demonstrate that the ALSV VIGS vector induces silencing of genes in quinoa beyond those involved in carotenoid biosynthesis, we functionally analyzed genes involved in betalain biosynthesis using the ALSV-VIGS system. There are two structural groups of betalains: the red-violet betacyanins, and the yellow betaxanthins (Polturak and Aharoni, 2018). Currently, *CYP76AD1* and *DODA1* are considered to be involved in the key steps of betalain biosynthesis (Hatlestad et al., 2012; Polturak et al., 2016; Imamura et al., 2018; Sheehan et al., 2020). Although *CqCYP76AD1* was previously implicated in betacyanin biosynthesis and shown to contribute to hypocotyl pigmentation (Imamura et al., 2018), it remains unknown whether *CqCYP76AD1* and *CqDODA1* are involved in betalain biosynthesis in whole quinoa plants.

**FIGURE 3:**
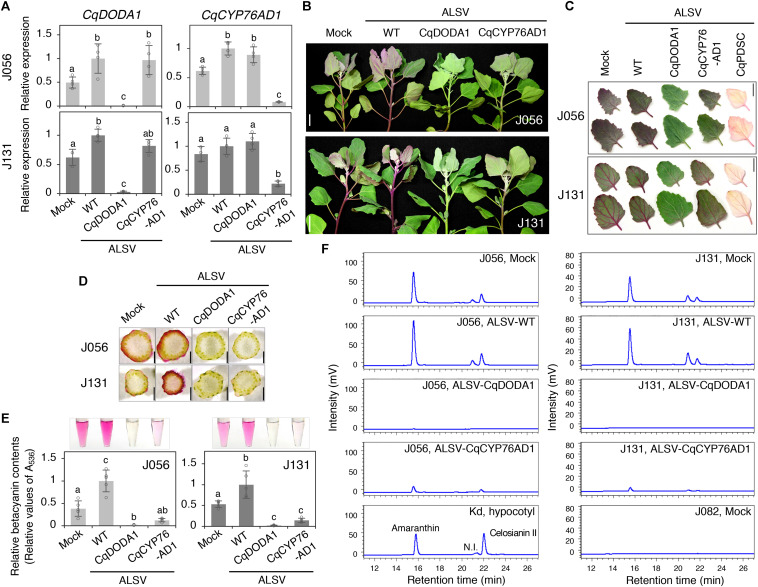
Silencing of *CqDODA1* and *CqCYP76AD1* inhibits betalain production in quinoa. **(A)** RT-qPCR analysis of *CqDODA1* and *CqCYP76AD1* transcripts in the uninoculated upper leaves of the inbred lines J056 and J131 inoculated with the indicated inocula. Data were normalized to quinoa *polyubiquitin 10* (*CqUBQ10*) expression and are shown as means ± SD (*n* = 4). Different letters indicate significant differences by a Tukey’s HSD test (*p* < 0.05). **(B)** Representative images of quinoa plants at 28 dpi with mock buffer, ALSV-WT, ALSV-CqDODA1, and ALSV-CqCYP76AD1. Scale bars represent 2 cm. **(C)** Representative images of the uninoculated upper leaves of quinoa plants at 20 dpi with the indicated inocula. Scale bars represent 2 cm. **(D)** Representative images of stem cross-sections of plants at 28 dpi with the indicated inocula. Scale bars represent 1 mm. **(E)** Relative quantification of betacyanin content was assessed spectrophotometrically by light absorption measurements at 536 nm. Relative values are presented as means ± SD (*n* = 5). Betacyanin pigments were extracted from the uninoculated upper leaves of quinoa plants (inbred J056 and J131 lines) at 16 dpi inoculated with the indicated inocula. Representative images of aqueous extracts used for the measurements are shown. **(F)** High performance liquid chromatography analysis of betacyanin pigments in the aqueous extracts used in **(E)**. The peak names of quinoa betacyanins composed of amaranthin, celosianin II, and a non-identified (N.I.) compound were identified based on the analysis of red-violet pigment extracted from hypocotyls of 14-day-old Kd plants (Imamura et al., 2019). No betacyanin peaks were detected in the uninoculated upper leaves of J082 inbred line plants inoculated with mock. The horizontal and vertical axes indicate the retention time (min) and signal intensity (mV), respectively.

To examine the possible roles of *CqCYP76AD1* and *CqDODA1* in the betalain biosynthesis pathway, VIGS was used to suppress gene expression in the quinoa inbred lines J056 and J131, which produce large amounts of betalains that can easily be seen under the experimental conditions (Figure 3 and Supplementary Figure 6). RT-qPCR analysis showed that the targeted genes were strongly silenced in the uninoculated upper leaves of both lines (Figure 3A). Silencing of *CqCYP76AD1* resulted in the reduction of red-violet batalain pigments in the uninoculated upper leaves and stems of both inbred lines (Figures 3B–D), whereas silencing of *CqDODA1* resulted in an almost complete loss of red-violet betalain pigments in the uninoculated upper leaves and stems in both lines (Figures 3B–D). By contrast, silencing of *CqPDS1* resulted in photobleaching phenotypes in the uninoculated upper leaves of both lines and we were thus able to confirm the accumulation of red-violet batalain pigments in the leaves (Figure 3C). The observed changes in pigment phenotype in *CqDODA1-* or *CqCYP76AD1*-silenced J056 and J131 lines were verified through spectrophotometric measurements showing the reduction of red-violet betacyanins in the uninoculated upper leaves (Figure 3E) and high-performance liquid chromatography (HPLC) analysis indicating the reduction of betacyanins composed of amaranthin, celosianin II, and an unidentified compound (Imamura et al., 2019; Figure 3F). These observations demonstrate that *CqCYP76AD1* and *CqDODA1* are required for the production of betacyanin pigments in the leaves and stems of the inbred lines J056 and J131, suggesting that these genes function in key steps of betalain biosynthesis in whole quinoa plants. Notably, we also established that ALSV-WT infection significantly upregulated the expression of at least *CqDODA1* in the inbred lines J056 and J131 (Figure 3A). This finding is consistent with the observation that ALSV-WT infection enhanced betalain accumulation in the leaves and stems of some northern highland lines such as J056, J071, J073, and J075, and the southern highland line J131 (Figures 3B–F and Supplementary Figure 6). These results suggest that ALSV infection induces defense responses, including the promotion of betalain accumulation, in these inbred lines.

### Knockdown of *CqRHT1* Causes an Overgrowth Phenotype in Quinoa

Plant morphology and architecture have been targeted as important agronomic traits for high productivity in crops (Boden and Østergaard, 2019; Eshed and Lippman, 2019). To illustrate that the ALSV VIGS vector can be used for functional genomics in quinoa beyond the genes involved in the production of secondary metabolites such as pigments, we examined the function of a height-regulating gene using the ALSV system. Height-regulating *GAI/RGA/RHT/D8* genes, whose mutations enabled the “Green Revolution” in wheat (*Triticum aestivum* L.), have been identified as negative regulators of gibberellin (GA) signaling and belong to the GRAS family of transcriptional regulators with DELLA domains (Peng et al., 1999; Ikeda et al., 2001; Olszewski et al., 2002; Hedden, 2003).

Therefore, we sought to identify the *GAI/RGA/RHT/D8* genes in quinoa. We obtained sequence data of quinoa orthologs of *GAI/RGA/RHT/D8* genes, named *CqRHT1*, from public databases, and designed PCR primers based on the conserved regions for cloning the genes. We identified two CDS of *CqRHT1* homoeologs, *CqRHT1A* and *CqRHT1B* (Supplementary Figure 9), in the standard quinoa inbred line Kd (Yasui et al., 2016). The cloned CDS sequence of *CqRHT1A* shared 97.9% identity with that of *CqRHT1B* (Supplementary Figure 9). A 300-bp fragment of the homoeolog *CqRHT1* in the highly conserved regions between these two genes was introduced into the ALSV-RNA2 vector to construct a ALSV RNA2 VIGS vector. We obtained inocula for VIGS analyses, including the packaged viruses of ALSV-CqRHT1, from the uninoculated upper leaves with chlorotic spots of quinoa plants (Iw line) inoculated with ALSV-RNA1 cDNA plasmid combined with ALSV-RNA2 VIGS vectors either harboring or not a specific 300-bp trigger sequence required for VIGS. To test whether *CqRHT1* influences plant morphology or architecture, we suppressed the expression of this gene using VIGS in the quinoa inbred line J082, which shows almost no symptoms and exhibited no negative effect on growth and plant size following inoculation with ALSV-WT (Table 1 and Supplementary Figure 2).

**FIGURE 4:**
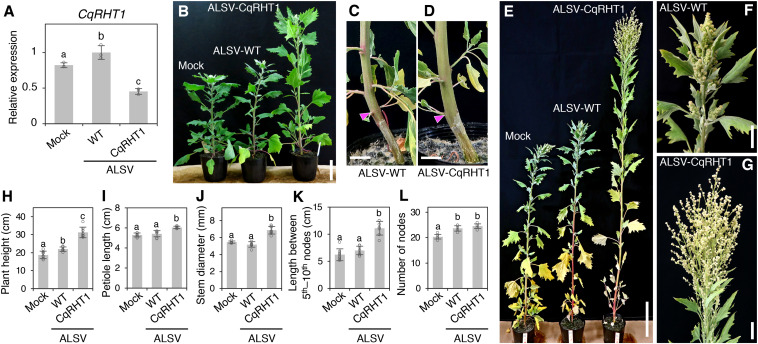
Knockdown of *CqRHT1* causes an overgrowth phenotype in quinoa. **(A)** RT-qPCR quantification of *CqRHT1* transcripts in the uninoculated upper leaves of the quinoa inbred line J082 inoculated with mock buffer, ALSV-WT, and ALSV-CqRHT1. Data were normalized to *CqUBQ10* expression and are shown as means ± SD (*n* = 3). **(B–G)** Representative images of plants inoculated with mock buffer, ALSV-WT, and ALSV-CqRHT1 at 21 **(B)**, 28 **(C,D)**, and 37 **(E–G)** dpi. Whole plants **(B,E)**, stem **(C,D)**, and inflorescence **(F,G)** are shown. Arrowheads in **(C)** and **(D)** indicate the position at the first node. Scale bars represent 5 cm **(B)**, 1 cm **(C,D)**, 10 cm **(E)**, or 2 cm **(F,G)**. **(H–L)** Statistical analysis of the growth of quinoa plants inoculated with mock buffer, ALSV-WT, and ALSV-CqRHT1. Plant height **(H)**, petiole length of the eighth leaf **(I)**, stem diameter at the first node **(J)**, internode length between the 5th and 10th nodes **(K)**, and number of nodes **(L)** were measured at 20 dpi. The data are presented as means ± SD (*n* = 8). Different letters in **(A,H–L)** indicate significant differences by a Tukey’s HSD test (*p* < 0.05).

RT-qPCR analysis showed that *CqRHT1* expression was significantly down-regulated in plants inoculated with ALSV-CqRHT1 compared with those inoculated with ALSV-WT (Figure 4A). Knockdown of *CqRHT1* significantly enhanced plant height, stem diameter, and petiole length, indicating that *CqRHT1* knockdown causes an overgrowth phenotype in quinoa (Figure 4 and Supplementary Figure 10). The enhanced plant height phenotype of plants inoculated with ALSV-CqRHT1 was due to the enhanced internode length rather than an increase in node number (Figures 4K,L and Supplementary Figures 10A,B). *CqRHT1* knockdown resulted in an abnormal inflorescence size and architecture with elongated branches (Figures 4E–G). These findings are in accordance with the previous report that loss-of-function mutations in *GAI/RGA/RHT/D8* genes caused an overgrowth phenotype in rice (*Oryza sativa* L.) and barley (*Hordeum vulgare* L.) (Ikeda et al., 2001; Chandler et al., 2002).

Furthermore, the *CqRHT1*-knockdown plants infected with ALSV-CqRHT1 showed enhanced cell division and elongation in the stem in comparison with the control plants infected with ALSV-WT (Supplementary Figures 10C–J). These results suggest that *CqRHT1* functions as a negative regulator of GA signaling in quinoa. In support of this, we confirmed that putative GA-responsive genes were up-regulated in the quinoa plants inoculated with ALSV-CqRHT1 in comparison with the plants inoculated with ALSV-WT (Supplementary Figure 10K). Taken together, these observations show that ALSV VIGS vectors can be used to alter plant morphology and architecture, and demonstrate that *CqRHT1* genes play a crucial role in controlling plant morphology and architecture through GA signaling in quinoa.

### ALSV VIGS Can Be Transmitted to the Progeny Plants Through Seeds at a Low Frequency

**FIGURE 5:**
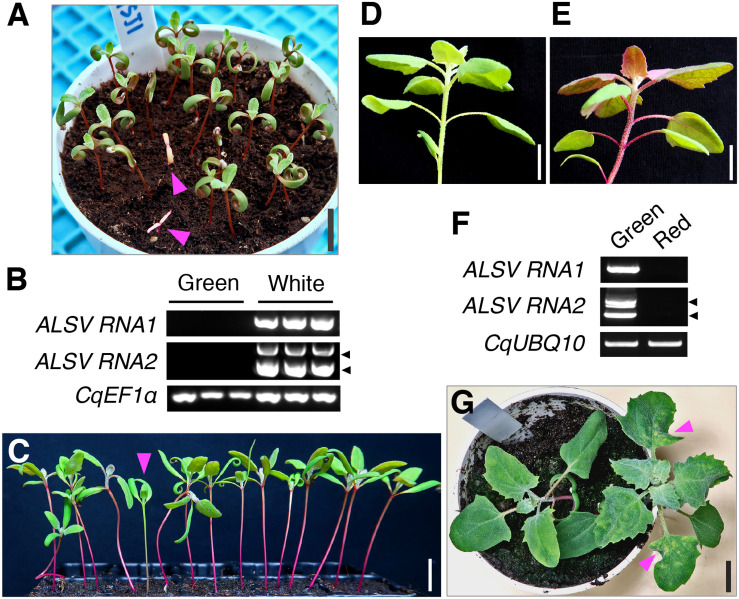
ALSV VIGS can be transmitted to progeny plants through seeds at a low frequency. **(A)** A representative image of progeny seedlings with the photobleaching phenotype at 7 dpg, derived from plants inoculated with ALSV-CqPDSC in the inbred Iw line. Arrowheads indicate progeny seedlings with the photobleaching phenotype. **(B)** Semi-quantitative RT-PCR of ALSV RNA1 and RNA2 in the progeny seedlings with (white) or without (green) the photobleaching phenotype shown in **(A)**. *CqEF1*α was used as an internal control. Arrowheads indicate the positions of recombinant ALSV RNA2 with the trigger sequence (top) and WT RNA2 without the trigger sequence (bottom). **(C)** An arrowhead indicates a progeny seedling that has not accumulated betalain in the hypocotyl at 14 dpg, derived from J056 inbred line plants inoculated with ALSV-CqDODA1. **(D,E)** Representative images of a plant with a green hypocotyl **(D)** or red hypocotyl **(E)** at 4 wpg, derived from J056 inbred line plants inoculated with ALSV-CqDODA1. **(F)** Semi-quantitative RT-PCR of ALSV RNA1 and RNA2 in progeny seedlings with a green or red hypocotyl shown in **(D,E)**. *CqUBQ10* was used as an internal control. Arrowheads indicate positions of recombinant ALSV RNA2 with the trigger sequence (top) and WT RNA2 without the trigger sequence (bottom). **(G)** A representative image of a progeny plant with chlorotic spots in leaves at 20 dpg, derived from the Iw inbred line plants inoculated with ALSV-WT. Arrowheads point to chlorotic spots in leaves in progeny seedlings with ALSV-WT. Scale bars represent 1 cm.

As ALSV can be transmitted to progeny plants through seeds from infected apple (*Malus domestica* Borkh.), *Nicotiana benthamiana* L., and soybean (*Glycine max* L. Merr.) plants (Yamagishi and Yoshikawa, 2009; Nakamura et al., 2011; Kishigami et al., 2014; Kamada et al., 2018), we next examined whether ALSV VIGS was inherited by the progeny of infected quinoa plants. In the Iw line, 0% (0/169) and 3.7% (17/449) of the progeny from the quinoa plants inoculated with ALSV-CqPDSN and ALSV-CqPDSC, respectively, displayed photobleaching in cotyledons and thus retained silencing (Figure 5A and Table 2). RT-PCR analysis confirmed that viral RNAs were present in the seedlings with white cotyledons but not in those with green cotyledons (Figure 5B). We also found that 1.7% (2/120) and 0% (0/120) of progeny from the quinoa plants inoculated with ALSV-CqDODA1 did not exhibit betalain accumulation in whole plants and thus retained silencing in the inbred lines, J056 and J131, respectively (Figures 5C–E and Table 2). Viral RNAs were detected in the seedlings that lacked betalain accumulation but not in those that accumulated betalain (Figure 5F). Thus, the seed transmission rates of VIGS were very low (less than 5%). By contrast, 12.1% (19/176) of the progeny of the Iw plants inoculated with ALSV-WT showed characteristic chlorotic spots in the leaves (Figure 5G and Table 2). These observations suggest that the seed transmission rate of ALSV in quinoa depends on whether or not a trigger sequence has been inserted into the VIGS vector and on the genotype of quinoa used.

**TABLE 2:** Summary of vertical transmission rates of ALSV in quinoa plants.

Inoculum	Name of inbred line	Visible symptom or phenotype	No. of progeny plants with visible phenotype/No. of total progeny plants
ALSV-WT	Iw	Chlorotic spots on the leaves	19/176 (12.1%)
ALSV-CqPDSN	Iw	Not detected	0/169 (0%)
ALSV-CqPDSC	Iw	Photobleaching of the cotyledons	17/449 (3.7%)
ALSV-CqDODA1	J056	Lack of betalain accumulation	2/120 (1.7%)
ALSV-CqDODA1	J131	Not detected	0/120 (0%)

## Discussion

Here, we show that ALSV-mediated transient expression techniques enable gene function studies in a wide variety of quinoa lines spanning all three sub-populations and can be used to alter the production of secondary metabolites such as pigments, carotenoids, and betalains, and also the plant morphology and architecture of quinoa. Although quinoa gene functions have been evaluated by the ectopic transient overexpression of exogenous genes in quinoa hairy roots, *Nicotiana benthamiana* leaves, and tobacco BY-2 suspension cells (Imamura et al., 2018, 2019, 2020), our findings presented here provide the quinoa research community with the gain- and loss-of-function tools needed to analyze the functions of endogenous genes *in planta* and enable functional genomics in quinoa.

Our results illustrate that ALSV VOX as well as VIGS can be used in quinoa (Supplementary Figure 8). However, since viruses with icosahedral virions, including ALSV, allow just small inserts due to size constraints for virus genome packaging (Abrahamian et al., 2020), ALSV can only be used to overexpress relatively small proteins such as FLOWERING LOCUS T and GFP (Takahashi and Yoshikawa, 2008; Yamagishi et al., 2014). There is thus a growing need to develop transformation and genome editing techniques that allow for the dissection of gene function in quinoa. We were intrigued to note that ALSV could be transmitted to the progeny plants via seeds from infected quinoa plants, although the transmission efficiency was low (0–10%), depending on the presence or absence of the insertion sequence and other factors such as quinoa lines and insertion sequences (Figure 5 and Table 2). The mechanism by which ALSV is able to enter the quinoa meristem is unclear, but this property could be exploited in future studies. Although this method is not normally applicable to the analysis of young seedlings, it is possible to analyze young seedlings in which a specific gene is knocked down, by using the progeny plants to which ALSV has been transmitted (Figure 5). Since growth was affected to a lesser extent in *CqDODA1*-silenced seedlings than in *CqPDS1*-silenced seedlings (Figure 5), *CqDODA1*-silenced plants could serve as a useful visible marker to evaluate the efficiency with which ALSV VIGS is transmitted to progeny plants under different experimental conditions.

Although betalains have attracted attention in recent years as plant pigments with promising health-promoting potential and visible fluorescence (Gandía-Herrero et al., 2016; Guerrero-Rubio et al., 2020), research on quinoa betalains has only recently begun. A previous mutant analysis indicated that *CqCYP76AD1* functions in betalain accumulation during the hypocotyl pigmentation process in quinoa (Imamura et al., 2018). Our VIGS results clearly extend the previous findings and show that *CqDODA1* and *CqCYP76AD1* play crucial roles in the production of betalain pigments accumulated in leaves and stems in quinoa (Figure 3). These findings support the notion that *CqDODA1* and *CqCYP76AD1* participate in key steps of betalain synthesis in whole quinoa plants. Recently, the function of quinoa *amaranthin synthetase 1* (*CqAMASY1*), which is involved in the synthesis of amaranthin, a major component of quinoa red-violet betacyanins, has been evaluated using an overexpression approach in tobacco BY-2 cultured cells (Imamura et al., 2019). ALSV VIGS would be useful to establish whether *CqAMASY1* and the other key genes involved in betalain synthesis function in allotetraploid quinoa as well. Plants of the Caryophyllales order, in which betalains occur in a mutually exclusive manner with anthocyanins (Polturak and Aharoni, 2018), are non-model plants and thus are not amenable to transformation in most cases. Therefore, viral vectors such as ALSV VIGS would be useful tools to decipher the biosynthesis pathways and evolution of betalain in Caryophyllales species.

The role of betalains in biotic stress responses in plants is poorly understood (Polturak and Aharoni, 2018). Our observations indicated that ALSV infection promotes betalain accumulation in quinoa (Figures 3B–F and Supplementary Figure 6), coupled with enhanced expression of betalain-related genes (Figure 3A). Based on previous reports of increased betalain synthesis induced by fungal and bacterial infections (Sepúlveda-Jiménez et al., 2004; Polturak et al., 2017), it is possible that the increase in betalain synthesis caused by ALSV infection may involve increased radical scavenging activity. The role of betalains in abiotic stress responses in quinoa is also not understood, even though quinoa is well adapted to harsh environments. A striking feature of the order Caryophyllales, to which quinoa belongs, is that its members are predominantly distributed in arid and semi-arid regions and in saline and alkaline soils (Polturak and Aharoni, 2018). The activity of betalains in defense against abiotic stress is likely mediated via their potent antioxidant capacity (Jain and Gould, 2015). With these considerations in mind, the role of betalains in abiotic and biotic stress responses in quinoa merits further investigation.

We show that *CqRHT1* knockdown causes an overgrowth phenotype in quinoa, characterized by increased plant and inflorescence height, stem diameter, and internode length (Figure 4 and Supplementary Figure 10), demonstrating that the ALSV VIGS vector can be used to alter the plant morphology and architecture of quinoa. Considering that mutations in the flower-promoting protein florigen and its antagonist antiflorigen or in the growth stimulating phytohormone GA-DELLA system could be used to reduce plant height and extend the geographical distribution in quinoa (Eshed and Lippman, 2019), the ALSV vector could contribute to research aimed at radically improving the productivity of quinoa. Indeed, in recent years, quinoa has been used as a model plant to explore the molecular mechanisms underlying abiotic stress tolerance in plants. Salt tolerance has been extensively studied with respect to the roles and functions of epidermal bladder cells (Shabala et al., 2014; Kiani-Pouya et al., 2017, 2019, 2020; Zou et al., 2017; Böhm et al., 2018), xylem ion loading (Zarei et al., 2020), tonoplast channels (Bonales-Alatorre et al., 2013), and inorganic ions for osmotic adjustment (Hariadi et al., 2011). Unlike rice and maize (*Zea mays* L.), which require high-light conditions to keep plants healthy, quinoa plants grow well in the growth chambers normally used for the model plant *Arabidopsis*. Therefore, plant researchers could easily shift their focus from the weedy model plant *Arabidopsis* to the new model plant quinoa, which has great practical value, and utilize the research resources that are rapidly being developed for this species (Yasui et al., 2016; Jarvis et al., 2017; Zou et al., 2017; Mizuno et al., 2020).

In conclusion, we have demonstrated that the ALSV vector for VIGS and VOX can be used for gene function analyses in a wide range of quinoa inbred lines from all three sub-populations. We further showed that silencing of *CqDODA1* and *CqCYP76AD1* inhibits betalain biosynthesis, that knockdown of *CqRHT1* results in an overgrowth phenotype in quinoa, and that ALSV is able to be transmitted to the progeny plants in quinoa. Thus, our findings enable functional genomics in quinoa, facilitating exploration of the molecular mechanisms of this mysterious plant with great environmental adaptability and outstanding nutritional profiles. Further, these techniques could be expected to contribute to the promotion of molecular breeding in quinoa.

## Materials and Methods

### Plant Materials and Growth Conditions

Seeds of quinoa (*Chenopodium quinoa* Willd.) inbred lines were generated previously (Yasui et al., 2016; Mizuno et al., 2020) and are listed in Table 1. The inbred line Kd is more stable in terms of phenotypic uniformity than the other lines, indicating that Kd is a suitable standard inbred line for molecular genetics and analyses (Yasui et al., 2016). The Iw line is used for propagation of ALSV (Mizuno et al., 2020). Quinoa plants were grown in soil in pots as described previously (Yasui et al., 2016; Mizuno et al., 2020), with minor modifications. Quinoa seeds were sown in a peat moss mixture (Jiffy Mix, Sakata Seeds, Yokohama, Japan) in a cell tray and were allowed to germinate in a growth chamber (Biotron LH-350S/410S: Nippon Medical & Chemical Instruments, Osaka, Japan) under controlled conditions of a 16-h light/8-h dark photoperiod, 22°C day/20°C night cycles, and a light intensity of 150 μmol photons m^–2^ s^–1^. After 7 days, the seedlings were transferred to a standard potting mix (Tsuchitaro, Sumitomo Forestry, Tokyo, Japan) in pots.

### Total RNA Isolation and RT-PCR Analysis

Total RNA isolation and complementary DNA (cDNA) synthesis were conducted essentially as described previously (Ogata et al., 2017). Total RNA was isolated from the uninoculated upper leaves of quinoa using RNAiso Plus (Takara Bio, Otsu, Shiga, Japan) according to the manufacturer’s instructions. Total RNA was pre-treated with RQ1 RNase-free DNase (Promega, Madison, WI, United States) and cDNA was synthesized using PrimeScript RT Master Mix (Takara Bio). Reverse transcription quantitative PCR (RT-qPCR) was performed using the QuantStudio 7 Flex real-time PCR system (Thermo Fisher Scientific, MA, United States) and TB Green Premix Ex Taq II (Takara Bio). Semiquantitative RT-PCR was performed using GoTaq Green Master Mix (Promega). The specific oligonucleotide primers used are listed in Supplementary Table 1.

### Molecular Cloning and Plasmid Construction

Based on the CDS and genome sequence for putative *CqPDS1* and *CqRHT1* obtained from public databases such as Quinoa Genome DataBase^1^ and Phytozome database^2^, using the amino acid sequences of *Arabidopsis* PDS (At4g14210) and wheat Rht-A1a (KC767924) as queries in the BLASTP program, we designed PCR primers (Supplementary Table 1) to determine the full-length CDS of the quinoa homologs in the standard quinoa inbred line Kd (Yasui et al., 2016). RT-PCR was performed using cDNA prepared from the inbred line Kd, amplified DNA fragments were inserted into the pGEM-T/Easy vector (Promega), and the multiple cloned sequences were determined by Sanger sequencing. We thus identified the homoeolog *CqPDS1* genes, *CqPDS1A* and *CqPDS1B*, and the homoeolog *CqRHT1* genes, *CqRHT1A* and *CqRHT1B*, in the inbred Kd line. The sequence data were deposited in the DDBJ/EMBL/GenBank database under the following accession numbers: *CqPDS1A* (LC591855); *CqPDS1B* (LC591856); *CqRHT1A* (LC591857); and *CqRHT1B* (LC591858). We designed PCR primers (Supplementary Table 1) to amplify trigger regions for VIGS (Supplementary Table 2) in the determined CDSs.

In contrast to *CqPDS1* and *CqRHT1*, multiple homologs of *CqDODA1* and *CqCYP76AD1* were identified in quinoa. We thus designed PCR primers that amplified trigger regions instead of sequencing the full-length *CqDODA1* and *CqCYP76AD1* genes. Based on the CDS of putative *CqDODA1* and *CqCYP76AD1* obtained from the public databases using *Mirabilis jalapa* DODA (AB435372) and *Beta vulgaris* CYP76AD1 (HQ656023) amino acid sequences as queries in the BLASTP program, we designed PCR primers (Supplementary Table 1) to amplify trigger regions for VIGS. RT-PCR was performed using cDNA from the Kd line and the amplified fragments were cloned into pEALSR2L5R5 (ALSV-RNA2 plasmid), which is the same as pER2L5R5XSB (Li et al., 2004; Supplementary Table 3). The cloned sequences were determined by Sanger sequencing (Supplementary Table 2). The cloned trigger sequences had 93% identity to *CqCYP76AD1-1* (XM_021913610) and 95.8% identity to *CqCYP76AD1-2* (XM_021876908), which were reported previously (Imamura et al., 2018).

ALSV-RNA2 vectors for VIGS analyses were constructed as described previously (Ogata et al., 2017), with minor modifications. The amplified DNA fragments containing trigger sequences of quinoa genes (Supplementary Table 2) for VIGS were cloned in-frame into the *Xho*I/*Bam*HI site of pEALSR2L5R5, generating pEALSR2-CqPDSN, pEALSR2-CqPDSC, pEALSR2-CqDODA1, pEALSR2-CqCYP76AD1, and pEALSR2-CqRHT1, respectively. To construct the ALSV-RNA2 vector for VOX analyses, *pGH-35S-sGFP* (Fujita et al., 2009) was digested with *Sma*I/*Eco*RV and the CDS of the *GFP* fragment was cloned into the *Sma*I site of pEALSR2L5R5, generating pEALSR2-sGFP. The resulting viruses derived from the ALSV-RNA2 constructs were designated ALSV-CqPDSN, ALSV-CqPDSC, ALSV-CqDODA1, ALSV-CqCYP76AD1, ALSV-CqRHT1, and ALSV-GFP-OE, respectively.

### Virus Inoculation

Quinoa plants were inoculated with ALSV as described previously (Ogata et al., 2017), with minor modifications. The plasmid DNAs (1–1.5 μg/μL) for the ALSV-RNA1 (pEALSR1) and each ALSV-RNA2 construct were mixed in equal amounts, and the DNA solution (5–10 μL) was mechanically rub-inoculated onto the true leaves of 12-day-old quinoa plants (inbred Iw line) using 600-mesh carborundum (Nakarai Tesque, Kyoto, Japan). Reverse osmosis (RO) water was used for mock inoculation. The inoculated quinoa plants were grown for 2–3 weeks and the uninoculated upper leaves showing chlorotic spots symptoms were harvested. The harvested leaves were grounded in extraction buffer (0.1 M Tris-HCl, pH 8.0, 0.1 M NaCl, 5 mM MgCl_2_) (Igarashi et al., 2009). Debris was precipitated by centrifugation at 18,800 g for 10 min at 4°C, and the supernatants were used as inocula for all quinoa inbred lines. The infected leaves or the inocula in extraction buffer were stored at –80°C before use. Seedlings (11–13 days old) of quinoa inbred lines of interest were mechanically rub-inoculated with the inocula (5–10 μL) using carborundum. ALSV derived from quinoa Iw plants inoculated with pEALSR1 and pEALSR2L5R5 was used as a control (ALSV-WT).

### Pigment Chemical Analysis

Betalain pigments were extracted at 16 dpi from the uninoculated upper leaves of quinoa plants inoculated with mock buffer, ALSV-WT, ALSV-CqCYP76AD1, and ALSV-CqDODA1. Leaf samples were frozen in liquid nitrogen and then stored at –80°C before use. The samples were powdered using a ShakeMaster (BMS-A20TP, BioMedical Science, Tokyo, Japan) and extracted in 0.1% (w/v) ascorbic acid (Hatlestad et al., 2015) at a ratio of 0.1 g of fresh leaf per 1 mL buffer at 4°C overnight. After centrifugation at 18,800 g for 10 min at 4°C, the extracts were filtered through a 0.45 μm membrane filter (Shimadzu, Kyoto, Japan).

The relative betacyanin contents were estimated spectrophotometrically based on absorbance at 536 nm (Imamura et al., 2019; Guerrero-Rubio et al., 2020). UV–Vis spectra were acquired using a DeNovix DS-11 (DeNovix, Wilmington DE, United States) spectrophotometer.

HPLC separation of betalain pigments was performed essentially as described previously (Imamura et al., 2019). A JASCO HPLC system (Jasco, Tokyo, Japan) equipped with a pump (PU-4185-Binary), an auto-sampler (AS-4150), a column oven (CO-4060), and a photodiode array detector (MD-4010) was used for analytical HPLC separations. Samples were separated on a C18 column (4.6 × 250 mm, Wakopak Handy ODS, Fujifilm Wako Pure Chemicals, Osaka, Japan), and linear gradients were run from 0% B to 45% B over a 45 min period using 0.05% trifluoroacetic acid (TFA) in water (solvent A) and 0.05% TFA in acetonitrile (solvent B) at a flow rate of 0.5 mL/min at 25°C, with elution being monitored by absorbance at 536 nm.

### VOX Analysis

Seeds of quinoa J082 plants, whose growth was not affected by ALSV infection, were surface-sterilized for 5 min in 1% (v/v) sodium hypochlorite solution and germinated on half-strength Murashige and Skoog (MS) salt medium supplemented with 1% (w/v) sucrose and 0.8% (w/v) agar at 22°C in the dark. At 2 dpg, the roots and hypocotyls of quinoa J082 plants were mechanically rub-inoculated with the packaged virus derived from recombinant ALSV harboring a full-length *GFP* CDS, using 600-mesh carborundum. The inoculated seedlings were incubated on MS agar plate at 22°C in the dark. GFP fluorescence was observed in the roots of quinoa plants at 10 dpi using a fluorescence stereoscopic microscope (SMZ25, Nikon, Tokyo, Japan) equipped with a dedicated digital camera DS-Fi3.

### Statistical Analysis

One-way ANOVA with Tukey’s HSD tests were performed using R software version 3.6.3 (R Core Team, 2020).

## Data Availability Statement

The datasets presented in this study can be found in online repositories. The names of the repository/repositories and accession number(s) can be found in the article/Supplementary Material.

## Author Contributions

HM, TT, YY, and YF conceived a series of quinoa studies. NY conceived ALSV studies and provided the virus materials. TO designed and performed the most of experiments. MT produced and managed quinoa inbred lines and prepared the seed samples. CY-O and YK performed the HPLC experiments. KF assisted expression analysis. KT and YY performed database search. TO and YF wrote the manuscript. MT, YN, and NY revised and edited manuscript. YF supervised the research. All authors contributed to the article and approved the submitted version.

## Conflict of Interest

KT, TT, and HM were employed by the company Actree Co. The authors declare that this study received funding from the company Actree Co. The funder had the following involvement in the study: a series of quinoa research concepts and database searches. The remaining authors declare that the research was conducted in the absence of any commercial or financial relationships that could be construed as a potential conflict of interest.

## Footnotes

1 http://quinoa.kazusa.or.jp/

2 https://phytozome.jgi.doe.gov/pz/portal.html#

## Supplementary Material

Supplementary Figure 1 Alignment of nucleotide sequences of *CqPDS1* homoeolog genes, *CqPDS1A* and *CqPDS1B*. Asterisks indicate conserved bases between the two homoeolog genes. Two 300-bp VIGS trigger sequences inserted into ALSV-CqPDSN and ALSV-CqPDSC vectors are highlighted in magenta and turquoise, respectively. The primer positions designed for RT-qPCR analysis are indicated with blue arrows.

Supplementary Figure 2 Three lowland inbred lines of quinoa, J028, J045, and J082, display almost no symptoms and show no negative growth effects following ALSV infection. **(A)** Side (left panels) and top (right panels) views of representative images of lowland line plants at 16 dpi with mock buffer and ALSV-WT. **(B)** Representative images of enlarged views of the uninoculated upper leaves of Kd, J079, and J082 inbred line plants at 16 dpi with mock and ALSV-WT. Scale bars represent 2 cm.

Supplementary Figure 3 Southern highland inbred lines of quinoa show mild growth retardation following ALSV infection. Side (left panels) and top (right panels) views of representative images of southern highland line plants at 16 dpi with mock buffer and ALSV-WT. Scale bars represent 2 cm.

Supplementary Figure 4 Some northern highland inbred lines exhibit growth retardation and visible symptoms following ALSV infection. **(A)** Side (left panels) and top (right panels) views of representative northern highland line plants at 16 dpi with mock buffer and ALSV-WT. ALSV-infected plants showed some growth retardation to varying degrees in the northern highland lines used in this study. **(B)** Enlarged views of representative uninoculated upper leaves of J064 and J074 inbred line plants at 16 dpi with mock and ALSV-WT. Scale bars represent 2 cm.

Supplementary Figure 5 ALSV can infect plants systemically in a variety of quinoa inbred lines. **(A–C)** Semi-quantitative RT-PCR of ALSV RNA1 in the uninoculated upper leaves of plants at 16 dpi with mock and ALSV-WT in 19 representative lines, including seven lowland **(A)**, six southern **(B)**, and six northern highland lines **(C)**. *CqUBQ10* was used as an internal control. No DNA indicates the absence of DNA template in PCR as a negative control. Two or three independent plants per inbred line were examined.

Supplementary Figure 6 ALSV infection facilitates betalain accumulation in some highland inbred lines. **(A,B)** Top (left panels) and side (right panels) views of representative plants inoculated with mock buffer and ALSV-WT at 21 dpi for the northern highland lines (J056, J071, J073, and J075) **(A)** and at 28 dpi for the southern highland line (J131) **(B)**. Scale bars represent 2 cm.

Supplementary Figure 7 Temperature conditions can alter disease symptoms and VIGS induction in ALSV-infected quinoa plants. **(A)** Representative images of plants of the inbred Iw line at 7, 10, and 14 dpi with mock buffer, ALSV-CqPDSC, and ALSV-WT. The plants were grown at 22°C or 25°C from 0 dpi. **(B)** A representative image of 18-day-old plants at 0 dpi. Scale bars represent 2 cm.

Supplementary Figure 8 ALSV can be used as a VOX vector in roots. **(A)** Representative images of GFP fluorescence in quinoa roots at 10 dpi with mock buffer and the packaged virus (ALSV-GFP-OE) derived from recombinant ALSV harboring a full-length *GFP* CDS. Scale bars represent 1 mm. TL indicates transmitted light microscopy images. **(B)** Semi-quantitative RT-PCR of ALSV RNA1 and RNA2 in roots at 3 wpi with the indicated inocula. *18S rRNA* was used as an internal control.

Supplementary Figure 9 Alignment of nucleotide sequences of *CqRHT1* homoeolog genes, *CqRHT1A* and *CqRHT1B*. Asterisks indicate conserved bases between the two homoeologs. The VIGS trigger sequence that was inserted into the ALSV-CqRHT1 vector is highlighted in magenta. The positions of primers used for RT-qPCR analysis are indicated with blue arrows.

Supplementary Figure 10 Knockdown of *CqRHT1* results in cell expansion and elongation phenotypes. **(A)** A representative image of stems of J082 inbred line plants at 25 dpi with ALSV-WT and ALSV-CqRHT1. Blue arrows indicate internode regions between the second and fifth nodes of the quinoa plants, illustrating the location of the longitudinal sections shown in **(B)**. Magenta arrows indicate the positions of the transverse sections **(C–J)** of the stems in the quinoa plants. **(B)** A representative image of longitudinal sections of the internode regions between the second and fifth nodes of the quinoa plants shown in **(A)**. Scale bar represents 1 cm. **(C–J)** Representative microscopy images of transverse sections of the stems of the plants shown in **(A)**, stained with 0.01% toluidine blue before observation. Scale bars represent 1 mm **(C,D,G,H)**, 0.5 mm **(E,I)**, and 0.1 mm **(F,J)**. Pi, pith; Co, collenchyma; Ph, phloem; Xy, xylem. **(K)** Putative gibberellin-responsive genes were upregulated in the uninoculated upper leaves of J082 inbred line plants inoculated with ALSV-CqRHT1. The relative abundance of transcripts in the uninoculated upper leaves of plants at 15 dpi with mock, ALSV-WT, and ALSV-CqRHT1 was quantified by RT-qPCR analysis. Data were normalized to *CqUBQ10* expression and are shown as means ± SD (*n* = 3). Different letters indicate significant differences by a Tukey’s HSD test (*p* < 0.05). To evaluate the expression of putative gibberellin-responsive genes in quinoa, *CqACS9*, *CqAUX22B*, *CqEXPA11*, *CqEXPB3*, *CqGAST1*, *CqPIF3*, *CqPRE4*, and *CqSAUR21*, primers for real time RT-qPCR were designed using the BLAST program in quinoa genome databases based on the nucleotide sequences of the corresponding *Arabidopsis thaliana* gibberellin-responsive gene homologs obtained from the NCBI database.

Supplementary Table 1 Primers used in this study.

Supplementary Table 2 Trigger sequences inserted into ALSV vectors.

Supplementary Table 3 Nucleotide sequences of plasmids pEALSR1 and pEALSR2L5R5.
