# Supplementary material for: Virus-Mediated Transient Expression Techniques Enable Functional Genomics Studies and Modulations of Betalain Biosynthesis and Plant Height in Quinoa
Source: Front Plant Sci. 2021 Mar 18;12:643499. doi: 10.3389/fpls.2021.643499 (PMC8014037; doi:10.3389/fpls.2021.643499)
Supplement: Supplementary Figure 1 — Alignment of nucleotide sequences of CqPDS1 homoeolog genes, CqPDS1A and CqPDS1B. Asterisks indicate conserved bases between the two homoeolog genes. Two 300-bp VIGS trigger sequences inserted into ALSV-CqPDSN and ALSV-CqPDSC vectors are highlighted in magenta and turquoise, respectively. The primer positions designed for RT-qPCR analysis are indicated with blue arrows. [file Data_Sheet_1.pdf]

```
              ***************************** ******************* **************************************************
CqPDS1A     1 ATGAGTCATCTTGGATATAGTTTTGCTACACAATCCACATCAAAATATGCTCTTTTGGGAAATTTGAGTAACCCCACTTCAGTTTCATCTGGGTGTAGTG 100
CqPDS1B     1 ATGAGTCATCTTGGATATAGTTTTGCTACCCAATCCACATCAAAATATGTTCTTTTGGGAAATTTGAGTAACCCCACTTCAGTTTCATCTGGGTGTAGTG 100

              ***** **** **************** ************************************ ***** *****************************
CqPDS1A   101 AATTTATGGGTAATTCTGTAAGAAATTGCAGTTTTAGCAAAGAATTTGGCAAAAAGCAAAGAATTGGGTACTCCCCATTAAAGGTTGTTTGTGTAGATTA 200
CqPDS1B   101 AATTTCTGGGCAATTCTGTAAGAAATTACAGTTTTAGCAAAGAATTTGGCAAAAAGCAAAGAATAGGGTATTCCCCATTAAAGGTTGTTTGTGTAGATTA 200

              ************  ************    ******************** *********************** *************************
CqPDS1A   201 TCCAAGGCCAGAGCTTGATAATACAGCG--AATTTCTTGGAAGCTGCTTACTTGTCTTCGACTTTTCGGAATTCTCCCCGTCCGAAGAAGCCATTAGAAG 298
CqPDS1B   201 TCCAAGGCCAGAAATTGATAATACAG--TCAATTTCTTGGAAGCTGCTTATTTGTCTTCGACTTTTCGGAATTCACCCCGTCCGAAGAAGCCATTAGAAG 298

              ********** *********************** ** **************************************************************
CqPDS1A   299 TTGTAATTGCCGGTGCCGGTTTGGCTGGTCTATCCACGGCAAAGTATTTAGCAGATGCAGGTCACAAACCCATATTGCTGGAAGCACGAGATGTTTTGGG 398
CqPDS1B   299 TTGTAATTGCTGGTGCCGGTTTGGCTGGTCTATCTACAGCAAAGTATTTAGCAGATGCAGGTCACAAACCCATATTGCTGGAAGCACGAGATGTTTTGGG 398

              ************ ******************************** ******************************************************
CqPDS1A   399 TGGGAAGATAGCGGCATGGAAAGACAAGGATGGGGACTGGTATGAAACAGGGCTACATATATTCTTTGGGGCTTATCCAAATGTACAGAATCTGTTTGGA 498
CqPDS1B   399 TGGGAAGATAGCAGCATGGAAAGACAAGGATGGGGACTGGTATGAGACAGGGCTACATATATTCTTTGGGGCTTATCCAAATGTACAGAATCTGTTTGGA 498

              ***** **********************************************************************************************
CqPDS1A   499 GAGCTAGGTATTAATGACCGATTACAATGGAAGGAGCACTCAATGATTTTTGCAATGCCTAGCAAGCCTGGTGAATTTAGCCGCTTTGATTTTGAAGAAG 598
CqPDS1B   499 GAGCTCGGTATTAATGACCGATTACAATGGAAGGAGCACTCAATGATTTTTGCAATGCCTAGCAAGCCTGGTGAATTTAGCCGCTTTGATTTTGAAGAAG 598

              ** *************************************************************************************************
CqPDS1A   599 TCCTGCCTGCACCATTAAATGGCATATGGGCAATCTTAAGGAATAATGAAATGCTTACCTGGCCAGAGAAAATCAAGTTTGCTATTGGTCTCTTGCCTGC 698
CqPDS1B   599 TCTTGCCTGCACCATTAAATGGCATATGGGCAATCTTAAGGAATAATGAAATGCTTACCTGGCCAGAGAAAATCAAGTTTGCTATTGGTCTCTTGCCTGC 698

              ****************************************************************************************************
CqPDS1A   699 AATGGTTGGTGGACAATCGTATGTTGAGGCACAAGATGGTTTGAGTGTCCAAGAGTGGATGAGGAAGCAAGGTGTACCTGATCGTGTAACTGATGAAGTA 798
CqPDS1B   699 AATGGTTGGTGGACAATCGTATGTTGAGGCACAAGATGGTTTGAGTGTCCAAGAGTGGATGAGGAAGCAAGGTGTACCTGATCGTGTAACTGATGAAGTA 798

              *********************************************************** ******************************** *******
CqPDS1A   799 TTTATTGCCATGTCAAAGGCATTGAACTTCATAAATCCTGATGAACTTTCGATGCAGTGTATCTTGATTGCTCTGAACCGATTTTTGCAGGAGAAGCATG 898
CqPDS1B   799 TTTATTGCCATGTCAAAGGCATTGAACTTCATAAATCCTGATGAACTTTCGATGCAGTGCATCTTGATTGCTCTGAACCGATTTTTGCAGGAAAAGCATG 898

              ******************************************************************************* ********************
CqPDS1A   899 GTTCCAAAATGGCTTTCCTGGATGGCAATCCTCCGGAAAGGCTGTGCATGCCTATTGTTGAGCATATAGAGTCACTTGGCGGTGAAGTGCGACTCAATTC 998
CqPDS1B   899 GTTCCAAAATGGCTTTCCTGGATGGCAATCCTCCGGAAAGGCTGTGCATGCCTATTGTTGAGCATATAGAGTCACTTGGTGGTGAAGTGCGACTCAATTC 998

              ****************************************************************************************** ******** 
CqPDS1A   999 TCGTCTTCAGAAGATAAAGTTAGCTCAGGATGGAACTGTAGATAGTTTTTTGCTAACTAATGGAAAAGAGGTTAGAGGAGATGCTTATGTCTCTGCTGCT 1098
CqPDS1B   999 TCGTCTTCAGAAGATAAAGTTAGCTCAGGATGGAACTGTAGATAGTTTTTTGCTAACTAATGGAAAAGAGGTTAGAGGAGATGCTTATGTATCTGCTGCC 1098

              *********************************** *********** ** *************************************************
CqPDS1A  1099 CCAGTCGACATCCTGAAGCTGCTTCTACCTGATGAATGGAAAGAAATATCGTACTTCAAAAAGTTGGATAAACTAGTAGGAGTTCCAGTCATTAATGTTC 1198
CqPDS1B  1099 CCAGTCGACATCCTGAAGCTGCTTCTACCTGATGAGTGGAAAGAAATTTCATACTTCAAAAAGTTGGATAAACTAGTAGGAGTTCCAGTCATTAATGTTC 1198

              ************* ****** *******************************************************************************
CqPDS1A  1199 ACATATGGTTTGACAGAAAATTGAAGAATACATACGACCATCTACTATTTAGCAGGAGTAATATTTTGAGTGTCTATGCTGATATGTCATTGACATGCAA 1298
CqPDS1B  1199 ACATATGGTTTGATAGAAAACTGAAGAATACATACGACCATCTACTATTTAGCAGGAGTAATATTTTGAGTGTCTATGCTGATATGTCATTGACATGCAA 1298

              ****** ******** ************************************** ********* ******************* ***************
CqPDS1A  1299 GGAATACTATGATCCAAATAAATCTATGTTGGAATTGGTATTTGCACCAGCAGAAGAATGGATTGCTCGCAGTGACACTGATATAATTGAGGCAACAATG 1398
CqPDS1B  1299 GGAATATTATGATCCGAATAAATCTATGTTGGAATTGGTATTTGCACCAGCAGAGGAATGGATTTCTCGCAGTGACACTGATATTATTGAGGCAACAATG 1398

              ****************************************************************************************************
CqPDS1A  1399 AAAGAACTTGCCAAACTTTTCCCTGACGAAATTGCAGCTGATGGGAGCAAGGCTAAGATCCTCAAATATCATGTCGTCAAGACTCCAAGGTCTGTTTATA 1498
CqPDS1B  1399 AAAGAACTTGCCAAACTTTTCCCTGACGAAATTGCAGCTGATGGGAGCAAGGCTAAGATCCTCAAATATCATGTCGTCAAGACTCCAAGGTCTGTTTATA 1498

              ******************************* ***************** ******** ** ************** *********** ***********
CqPDS1A  1499 AGACAGTTCCAAATTGTGAACCTTGTCGGCCGCTGCAAAGATCACCTATAGAGGGTTTTTATTTATCTGGTGATTTCACGAAACAAAAATATTTGGCTTC 1598
CqPDS1B  1499 AGACAGTTCCAAATTGTGAACCTTGTCGGCCACTGCAAAGATCACCTATTGAGGGTTTCTACTTATCTGGTGATTTTACGAAACAAAAGTATTTGGCTTC 1598

              ********************************************************* ** *** *** ****** ***************** ** ** 
CqPDS1A  1599 AATGGAAGGAGCTGTTTTATCTGGGAAGTTTTGTGCACAGGCTATTGTGCAGGATTACGAGTTGCTTGCTGCTCGAGCACAAAGAGAATTGGCCGGAGCT 1698
CqPDS1B  1599 AATGGAAGGAGCTGTTTTATCTGGGAAGTTTTGTGCACAGGCTATTGTGCAGGATTATGAATTGATTGTTGCTCGTGCACAAAGAGAATTGGCAGGGGCG 1698

              ********** *
CqPDS1A  1699 GGCAATGTATGA 1710
CqPDS1B  1699 GGCAATGTATAA 1710
```

Figure S1 Alignment of nucleotide sequences of *CqPDS1* homoeolog genes, *CqPDS1A* and *CqPDS1B*. Asterisks indicate conserved bases between the two homoeolog genes. Two 300-bp VIGS trigger sequences inserted into ALSV-CqPDSN and ALSV-CqPDSC vectors are highlighted in magenta and turquoise, respectively. The primer positions designed for RT-qPCR analysis are indicated with blue arrows.

Ogata et al. Supplementary Figure S1

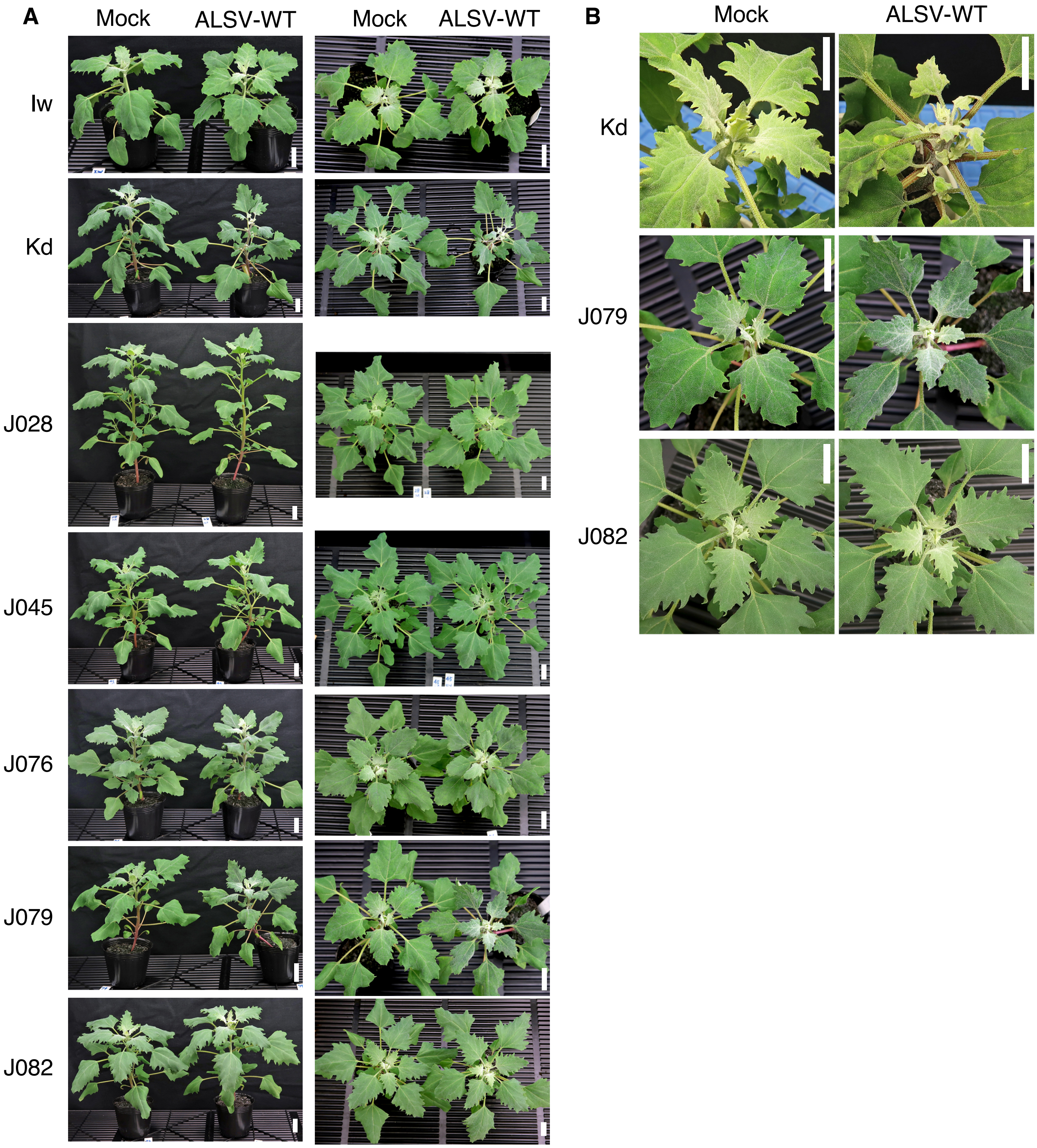

Figure S2 Three lowland inbred lines of quinoa, J028, J045, and J082, display almost no symptoms and show no negative growth effects following ALSV infection. (A) Side (left panels) and top (right panels) views of representative images of lowland line plants at 16 dpi with mock buffer and ALSV-WT. (B) Representative images of enlarged views of the uninoculated upper leaves of Kd, J079, and J082 inbred line plants at 16 dpi with mock and ALSV-WT. Scale bars represent 2 cm.

Ogata et al. Supplementary Figure S2

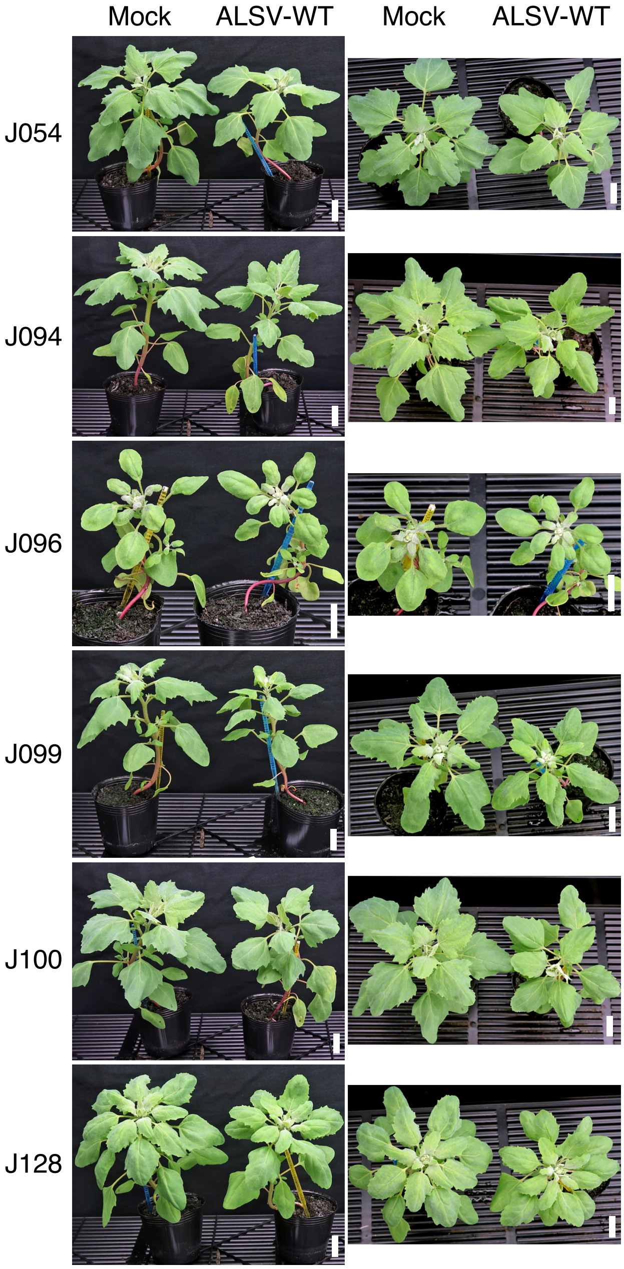

Figure S3 Southern highland inbred lines of quinoa show mild growth retardation following ALSV infection. Side (left panels) and top (right panels) views of representative images of southern highland line plants at 16 dpi with mock buffer and ALSV-WT. Scale bars represent 2 cm.

Ogata et al. Supplementary Figure S3

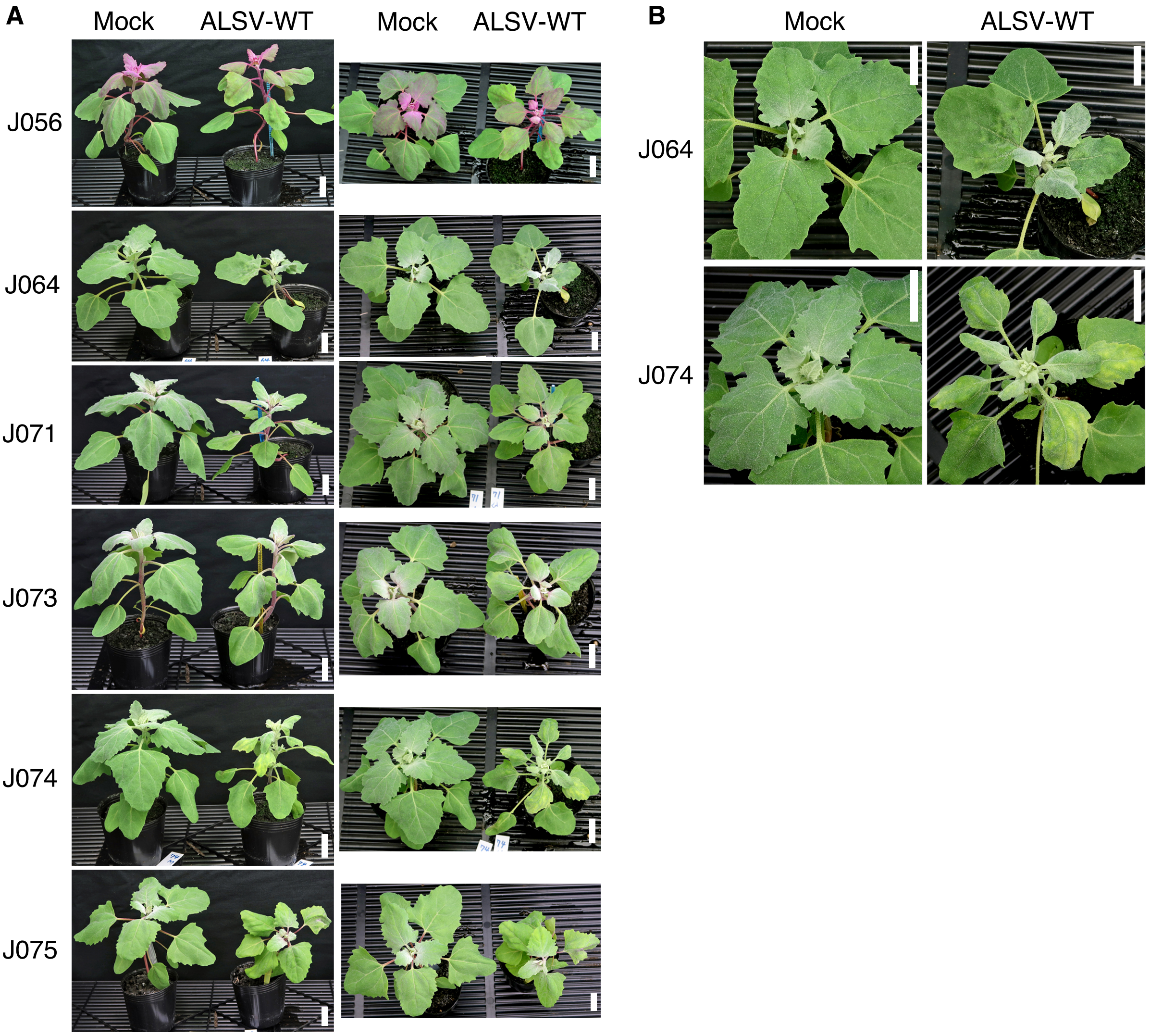

Figure S4 Some northern highland inbred lines exhibit growth retardation and visible symptoms following ALSV infection. (A) Side (left panels) and top (right panels) views of representative northern highland line plants at 16 dpi with mock buffer and ALSV-WT. ALSV-infected plants showed some growth retardation to varying degrees in the northern highland lines used in this study. (B) Enlarged views of representative uninoculated upper leaves of J064 and J074 inbred line plants at 16 dpi with mock and ALSV-WT. Scale bars represent 2 cm.

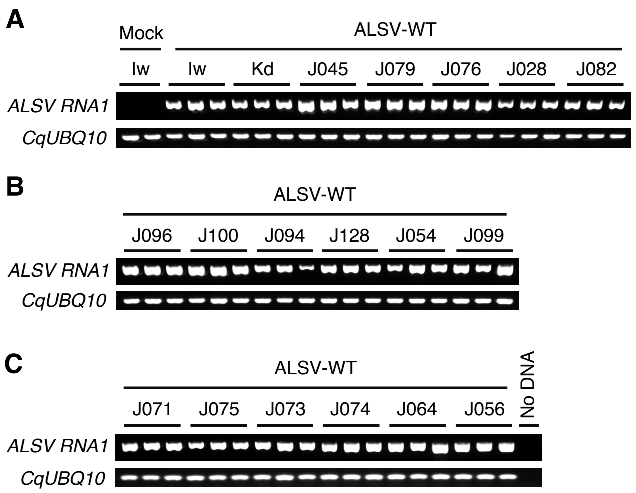

Figure S5 ALSV can infect plants systemically in a variety of quinoa inbred lines. (A–C) Semi-quantitative RT-PCR of ALSV RNA1 in the uninoculated upper leaves of plants at 16 dpi with mock and ALSV-WT in 19 representative lines, including seven lowland (A), six southern (B), and six northern highland lines (C). *CqUBQ10* was used as an internal control. No DNA indicates the absence of DNA template in PCR as a negative control. Two or three independent plants per inbred line were examined.

Ogata et al. Supplementary Figure S5
