# Supplementary material for: Virus-Mediated Transient Expression Techniques Enable Functional Genomics Studies and Modulations of Betalain Biosynthesis and Plant Height in Quinoa
Source: Front Plant Sci. 2021 Mar 18;12:643499. doi: 10.3389/fpls.2021.643499 (PMC8014037; doi:10.3389/fpls.2021.643499)
Supplement: Supplementary Figure 6 — ALSV infection facilitates betalain accumulation in some highland inbred lines. (A,B) Top (left panels) and side (right panels) views of representative plants inoculated with mock buffer and ALSV-WT at 21 dpi for the northern highland lines (J056, J071, J073, and J075) (A) and at 28 dpi for the southern highland line (J131) (B). Scale bars represent 2 cm. [file Data_Sheet_2.pdf]

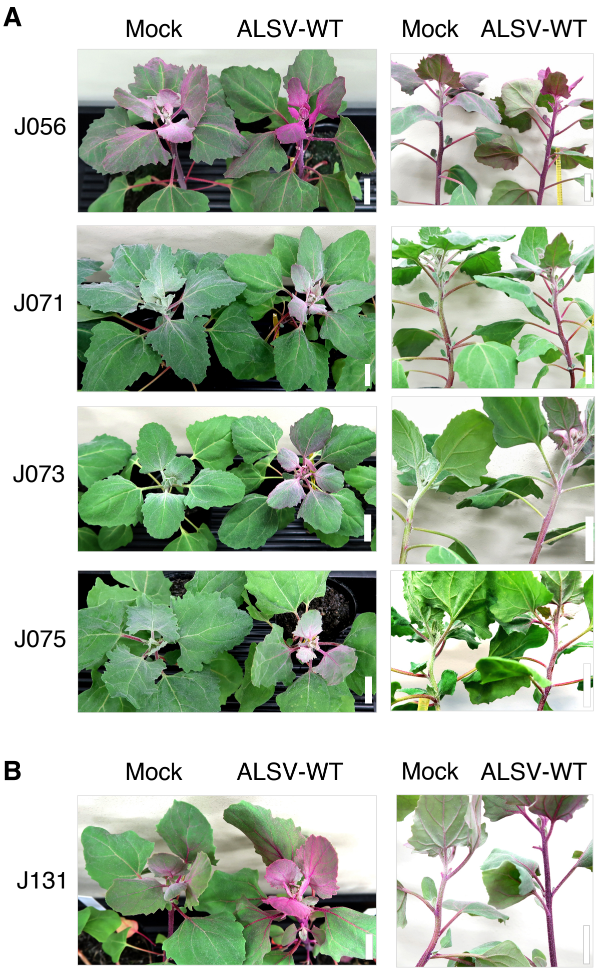

Figure S6 ALSV infection facilitates betalain accumulation in some highland inbred lines. (A–B) Top (left panels) and side (right panels) views of representative plants inoculated with mock buffer and ALSV-WT at 21 dpi for the northern highland lines (J056, J071, J073, and J075) (A) and at 28 dpi for the southern highland line (J131) (B). Scale bars represent 2 cm.

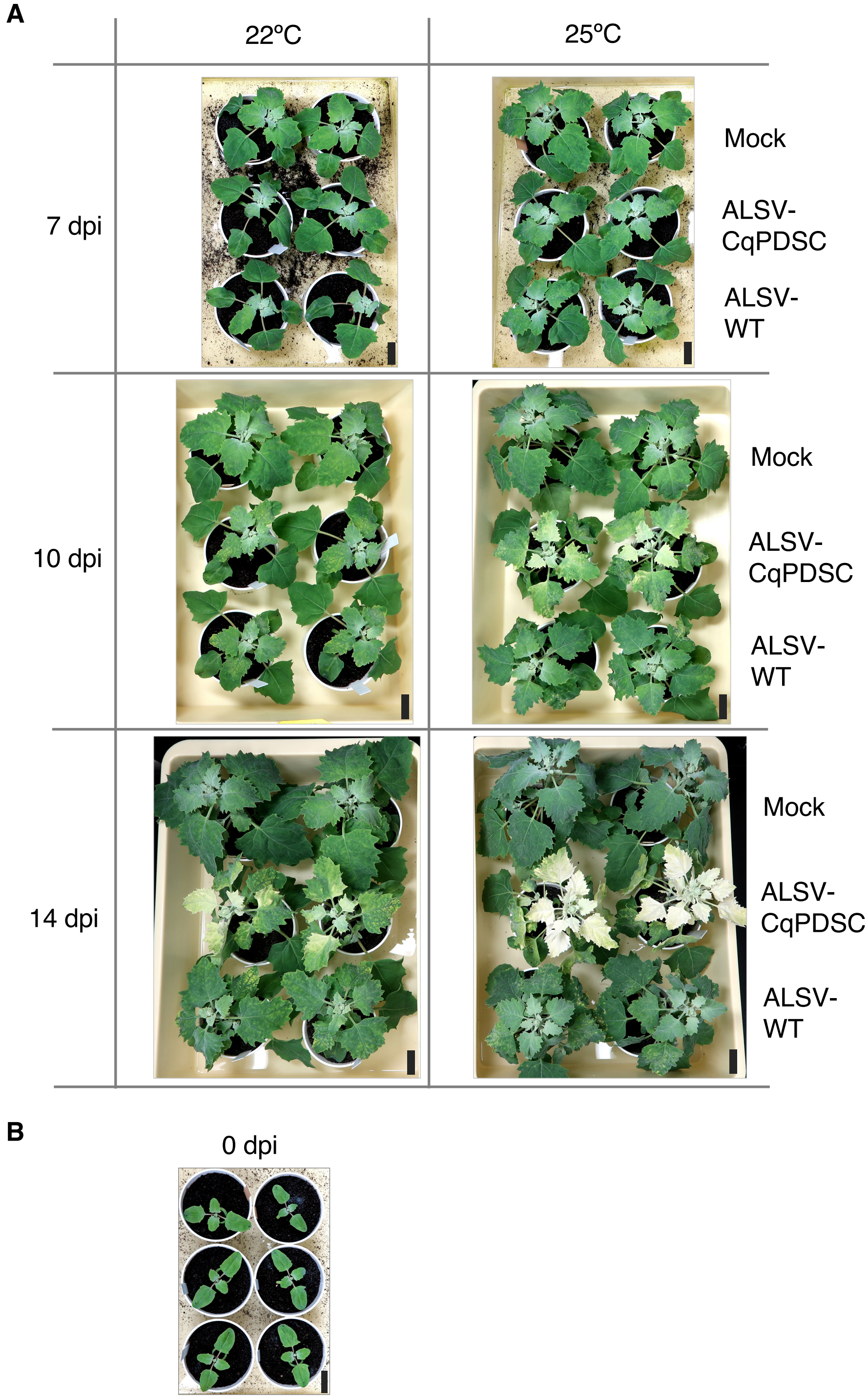

Figure S7 Temperature conditions can alter disease symptoms and VIGS induction in ALSV-infected quinoa plants. (A) Representative images of plants of the inbred Iw line at 7, 10, and 14 dpi with mock buffer, ALSV-CqPDSC, and ALSV-WT. The plants were grown at 22°C or 25°C from 0 dpi. (B) A representative image of 18-day-old plants at 0 dpi. Scale bars represent 2 cm.

Ogata et al. Supplementary Figure S7

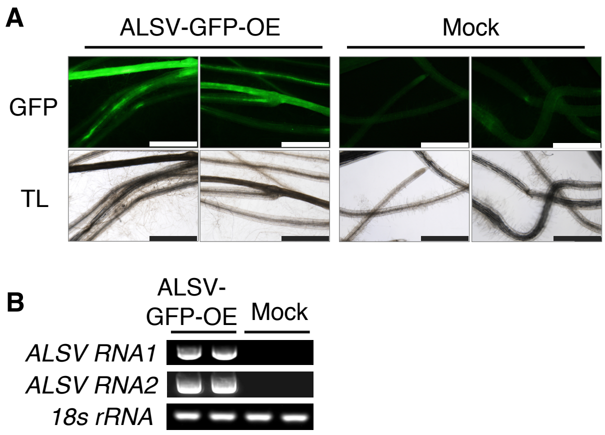

Figure S8 ALSV can be used as a VOX vector in roots. (A) Representative images of GFP fluorescence in quinoa roots at 10 dpi with mock buffer and the packaged virus (ALSV-GFP-OE) derived from recombinant ALSV harboring a full-length *GFP* CDS. Scale bars represent 1 mm. TL indicates transmitted light microscopy images. (B) Semi-quantitative RT-PCR of ALSV RNA1 and RNA2 in roots at 3 wpi with the indicated inocula. *18S rRNA* was used as an internal control.

```
               ************** ********************************************************************************
CqRHT1A      1  ATGAAGAGGGAACTACCCTTTACGAATCCAGAACCTTCTACCGGCGGTAAACCGCCGGTTTCTGGAAAGTCAAAGATGTGGGAAGATCAAAACGACGCCG  100
CqRHT1B      1  ATGAAGAGGGAACTCCCCTTTACGAATCCAGAACCTTCTACCGGCGGTAAACCGCCGGTTTCTGGAAAGTCAAAGATGTGGGAAGATCAAAACGACGCCG  100

               ****************************************************************************************************
CqRHT1A    101  GAGTTGATGAGTTGCTTGCCGTTTTGGGGTATAAGGTCAGGTCATCAGATATGGCGGAAGTTGCTCAGAAACTTGAACAGCTTGAACAAGCTATGGGAAA  200
CqRHT1B    101  GAGTTGATGAGTTGCTTGCCGTTTTGGGGTATAAGGTCAGGTCATCAGATATGGCGGAAGTTGCTCAGAAACTTGAACAGCTTGAACAAGCTATGGGAAA  200

               ****************************************************************************************************
CqRHT1A    201  TGTTCGAGAAGATGGTCTTTCTCAATTAGCTTCTGAAACTGTTCATTATAATCCTTCAGATCTGTCTACTTGGCTTGAATCTATGCTTTCTGAGTTTAAT  300
CqRHT1B    201  TGTTCGAGAAGATGGTCTTTCTCAATTAGCTTCTGAAACTGTTCATTATAATCCTTCAGATCTGTCTACTTGGCTTGAATCTATGCTTTCTGAGTTTAAT  300

               ** * ************ ******   *************** ********** *********************************************
CqRHT1A    301  CCTTGTACTACTTTTGACGATGATTCGTCGTCAAGTCAGATTTTATTACCTTCACCGATTGGGTCGGTTGTAGATCCGATTGTTAACCCGACTCCTCAGA  400
CqRHT1B    301  CCATCTACTACTTTTGATGATGAT---TCGTCAAGTCAGATTCTATTACCTTCTCCGATTGGGTCGGTTGTAGATCCGATTGTTAACCCGACTCCTCAGA  397

               ***** ****************************************** ***** ************** ********* ************ **********
CqRHT1A    401  GTTCGATCCGGTTCAGTTCCGACCCGTATTCGGATTATGATCTCAAGGCTATCCCAGGTAAAGCTATCTTGACTCCGCCTTCGCCGTCTTCTTCATCAGC  500
CqRHT1B    398  GTTCGGTCCGGTTCAGTTCCGACCCGTATTCGGATTATGATCTCAAAGCTATTCCAGGTAAAGCTATTTTGACTCCACCTTCGCCGTCTACTTCATCAGC  497

               ** **************** ********************************** ********************************************
CqRHT1A    501  TTCACTTGTCAATAACAATGATAATAATAACAATAATAGTAGTAGTTGTAATAGTTTGGTTAGTAATACTACAAGCAGCTCAAGTTCAAGAGAGGCTAAG  600
CqRHT1B    498  TTTACTTGTCAATAACAATAATAATAATAACAATAATAGTAGTAGTTGTAATAGTATGGTTAGTAATACTACAAGCAGCTCAAGTTCAAGAGAGGCTAAG  597

               ********************** *********** ******** ** ****************************************** ***** *
CqRHT1A    601  AGGTTGAAGGCCTCTAATTATACTGCTGCTAGTGCTACCGCTACCGCTACCGTCTCGAACCCGTCCTCATCGAAGTTAACCGCCAATTCAGCGGTGTCGA  700
CqRHT1B    598  AGGTTGAAGGCCTCTAATTATACGGCTGCTAGTGCAACCGCTACTGCAACCGTCTCGAACCCGTCCTCATCGAAGTTAACCGCCAATTCAGCCGTGTCAA  697

               **********  ************* ***** ***********************************************************************
CqRHT1A    701  GGCCGGTTGTTCTTGTTGATTCGCAAGAGAACGGTGTTAGATTGGTGCATACTTTGATGGCTTGTGCTGAGGCAATTGAGCAACAGAATATGGGATTAGC  800
CqRHT1B    698  GGCCGGTTGTGGTTGTTGATTCGCAGGAGAATGGTGTTAGATTGGTGCATACTTTGATGGCTTGTGCTGAGGCAATTGAGCAACAGAATATGGGATTAGC  797

               ********** ********************************** ******************* **********************************
CqRHT1A    801  TGAAGCGCTGTTAAAACAAATCGGGTTTTTAGCGGCGTCTCAAGTCGGATCTATGAGAAAGGTTGCTACTTATTTTGCTGAGGCTCTTGCAAGGAGGGTG  900
CqRHT1B    798  TGAAGCGCTGCTAAAACAAATCGGGTTTTTAGCGGCGTCTCAAGTTGGATCTATGAGAAAGGTTGCCACTTATTTTGCTGAGGCTCTTGCAAGGAGGGTG  897

               ************************************************ *********** ********* ****************************
CqRHT1A    901  TATAAATTATGCCCAGATGTTCCATATGATGGCAATTTATCAGATATGTTACAGATGCACTTCTACGAGACATGCCCCTATCTCAAATTCGCTCATTTCA  1000
CqRHT1B    898  TATAAATTATGCCCAGATGTTCCATATGATGGCAATTTATCAGATATGTTGCAGATGCACTTTTACGAGACTTGCCCCTATCTCAAATTCGCTCATTTCA  997

               **************************** ***********************************************************************
CqRHT1A   1001  CGGCGAATCAGGCGATTTTGGAGGCTTTCAGTGGCAAAAAGAAGGTTCATGTTATTGATTTCAGTATGAAAGAAGGGATGCAATGGCCAGCTTTGATGCA  1100
CqRHT1B    998  CGGCGAATCAGGCGATTTTGGAGGCTTTTAGTGGCAAAAAGAAGGTTCATGTTATTGATTTCAGTATGAAAGAAGGGATGCAATGGCCAGCTTTGATGCA  1097

               *** ** ********** ********* ******** ******** *********** ************** ***************************
CqRHT1A   1101  AGCATTAGCGCTTCGCCGTGAAGGCCCTCCGGCTTTCCGATTAACTGGGATTGGACCGCCAGCACCGGATAACTCGGACAGATTGCAAGAAGTTGGTTGG  1200
CqRHT1B   1098  AGCGTTGGCGCTTCGCCCTGAAGGCCCGCCGGCTTTTCGATTAACCGGATTGGACCACCAGCACCGGATAATTCGGACAGATTGCAAGAAGTTGGTTGG  1197

               **************** ** ******************************************* ************** ****** ********** *
CqRHT1A   1201  AAATTAGCTCAATTTGCTGACTCAATCCAAATCCAATTCGAATACCGTGGGTTTGTGGCCAATAGTTTGACCGATTTGGAGTCAGCCTTGCTTGATCTCA  1300
CqRHT1B   1198  AAATTAGCTCAATTTGCAGATTCAATCCAAATCCAATTCGAATACCGTGGGTTTGTGGCCAATAGCTTGACCGATTTGGAATCAGCCCTGCTTGATCTAA  1297

               ******* ********************** ** *** *********** *********** *************************************
CqRHT1A   1301  GACCCGAGACCGAAGTTGTCGCGGTTAATTCGGTGTTT-GAGCTGCATCGGTTGCTAGCTCGGCCCGGCGCAGCCGAAAAGGTGATGGGTTTAATGAAGG  1399
CqRHT1B   1298  GACCCGAAACCGAAGTTGTCGCGGTTAATTCAGT-TTTCGAGCTGCATCGTTTGCTAGCTCGCCCCGGCGCAGCCGAAAAGGTGATGGGTTTAATGAAGG  1396

               **** ************************************** ********    **** ********    ****************************
CqRHT1A   1400  AGGTTAACCCAGTCATAGTAACAGTAGTCGAGCAAGAGGCGAACCACAACGGAC--CGGCATTCCTAGACA--GGTTCAACGAATCACTCCACTACTACT  1495
CqRHT1B   1397  AGGTGAACCCAGTCATAGTAACAGTAGTCGAGCAAGAGGCGAATCACAACGG--GTCGGCTTTCCTAGA--TCGGTTCAACGAATCACTCCACTACTACT  1492

               ******************************************* ********************************************************
CqRHT1A   1496  CAACCCTGTTCGACTCCTTAGAGAGCTGCGTAGACAGCCAGGACAAGATGATGTCTGAAGTATACTTAGGGAGGCAAATATGCAACGTTGTTGCTTGTGA  1595
CqRHT1B   1493  CAACCCTGTTCGACTCCTTAGAGAGCTGCGTAGACAGCCAGGATAAGATGATGTCTGAAGTATACTTAGGGAGGCAAATATGCAACGTTGTTGCTTGTGA  1592

               ****** ********************** ************************** ************** *********** *********** **
CqRHT1A   1596  AGGAGTTGACCGAGTCGAACGCCACGAGACGCTGGCTCAATGGAGGAATCGGTTCGGATCAGCCGGGTTCGCTCCGGTCCATATTGGCTCAAACGCCTTT  1695
CqRHT1B   1593  AGGAGTCGACCGAGTCGAACGCCACGAGACACTGGCTCAATGGAGGAATCGGTTCGGAACAGCCGGGTTCGCGCCGGTCCATATCGGCTCAAACGCATTC  1692

               ***** ** ********     ******************************** ***** ***************************************
CqRHT1A   1696  AAACAAGCCAGTGTATTAT--TGGACTACTTTGCAGGCGGTGATGGGTATGGTGTTGAGGAGAATGATGGGTGTCTAATGTTGGGGTGGCATAGTAGGCC  1793
CqRHT1B   1693  AAACAGGCTAGTGTATT--GCTGGACTACTTTGCAGGCGGTGATGGGTATGGTGTGGAGGAAAATGATGGGTGTCTAATGTTGGGGTGGCATAGTAGGCC  1790

               *** ********************************************** **************
CqRHT1A   1794  ACTAATCACAACCTCAGCTTGGCAACTCGCCAAGAATAACCAAAACTCGACAACTCGGCTCTGA     1793
CqRHT1B   1791  ACTGATCACAACCTCAGCTTGGCAACTCGCCAAGAATAACCAAAACTCGCCAACTCGGCTCTGA     1790
```

Figure S9 Alignment of nucleotide sequences of *CqRHT1* homoeolog genes, *CqRHT1A* and *CqRHT1B*. Asterisks indicate conserved bases between the two homoeologs. The VIGS trigger sequence that was inserted into the ALSV-CqRHT1 vector is highlighted in magenta. The positions of primers used for RT-qPCR analysis are indicated with blue arrows.

Ogata et al. Supplementary Figure S9

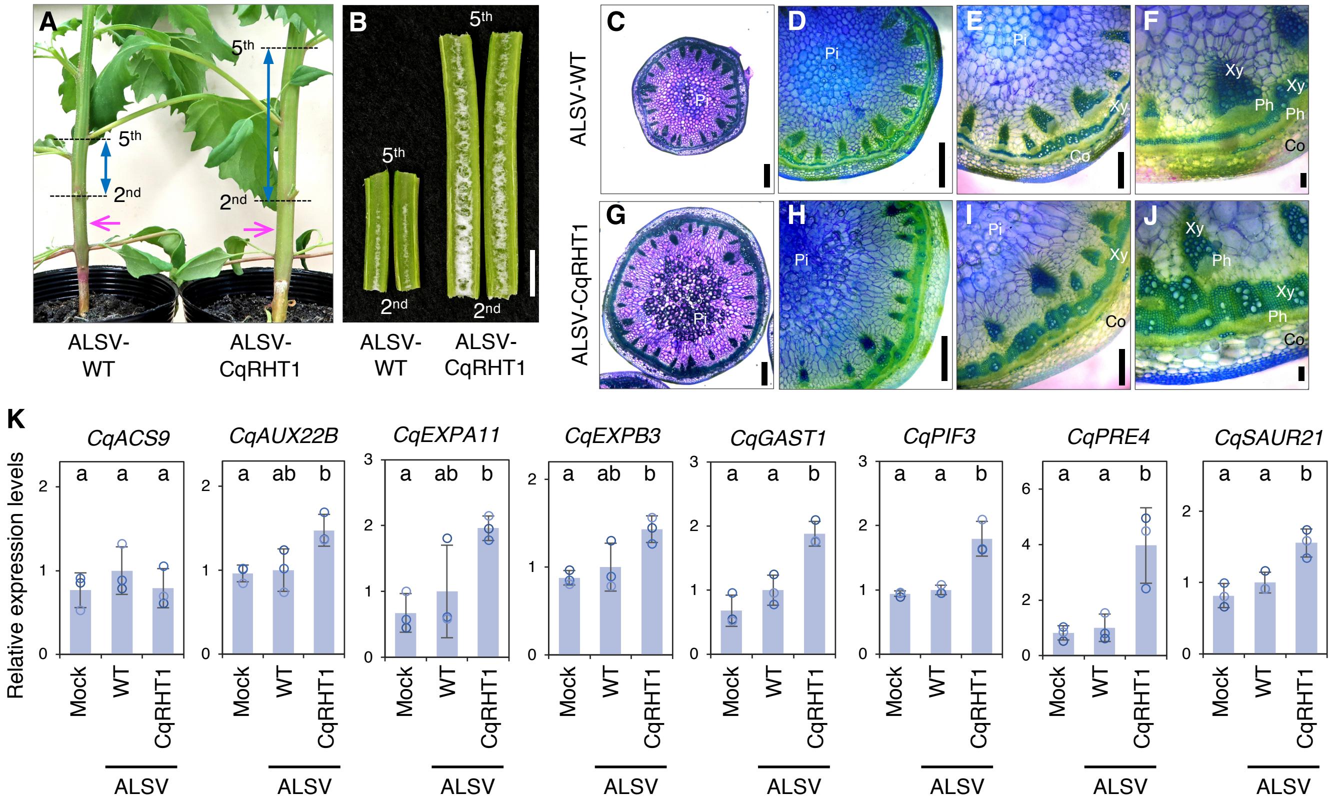

Figure S10 Knockdown of *CqRHT1* results in cell expansion and elongation phenotypes. (A) A representative image of stems of J082 inbred line plants at 25 dpi with ALSV-WT and ALSV-CqRHT1. Blue arrows indicate internode regions between the second and fifth nodes of the quinoa plants, illustrating the location of the longitudinal sections shown in **B**. Magenta arrows indicate the positions of the transverse sections (C–J) of the stems in the quinoa plants. (B) A representative image of longitudinal sections of the internode regions between the second and fifth nodes of the quinoa plants shown in **A**. Scale bar represents 1 cm. (C–J) Representative microscopy images of transverse sections of the stems of the plants shown in **A**, stained with 0.01% toluidine blue before observation. Scale bars represent 1 mm (C,D,G,H), 0.5 mm (E,I), and 0.1 mm (F,J). Pi, pith; Co, collenchyma; Ph, phloem; Xy, xylem. (K) Putative gibberellin-responsive genes were upregulated in the uninoculated upper leaves of J082 inbred line plants inoculated with ALSV-CqRHT1. The relative abundance of transcripts in the uninoculated upper leaves of plants at 15 dpi with mock, ALSV-WT, and ALSV-CqRHT1 was quantified by RT-qPCR analysis. Data were normalized to *CqUBQ10* expression and are shown as means ± SD (n = 3). Different letters indicate significant differences by a Tukey's HSD test ($p < 0.05$). To evaluate the expression of putative gibberellin-responsive genes in quinoa, *CqACS9*, *CqAUX22B*, *CqEXPA11*, *CqEXPB3*, *CqGAST1*, *CqPIF3*, *CqPRE4*, and *CqSAUR21*, primers for real time RT-qPCR were designed using the BLAST program in quinoa genome databases based on the nucleotide sequences of the corresponding *Arabidopsis thaliana* gibberellin-responsive gene homologs obtained from the NCBI database.
